# Yellowstone plume drives drainage reorganization in the early Miocene

**DOI:** 10.1126/sciadv.adz4275

**Published:** 2025-11-19

**Authors:** Dieke Gerritsen, Stuart A. Gilder, Yi-Wei Chen, Michael R. Wack

**Affiliations:** ^1^ Department of Earth and Environmental Sciences, Ludwig-Maximilians-Universität München, Theresienstrasse 41, 80333 Munich, Germany.; ^2^ Department of Geosciences, National Taiwan University, No. 1, Section 4, Roosevelt Road, Da’an District, Taipei City, 10617, Taiwan.; ^3^ Department of Earth and Atmospheric Science, Technische Universität München, Arcisstrasse 21, 80333 Munich, Germany.

## Abstract

The rise of the Yellowstone plume coincided with a shift from orogenic collapse of the Cordillera to Basin and Range extension in the northern Rocky Mountains, yet its impact on regional uplift, sediment dispersal, and drainage patterns remains largely unexplored. Here, we present rock magnetic analyses from eight Oligo-Miocene sedimentary sections in southwestern Montana. Using two complementary datasets, we identify a sharp increase in magnetic mineral concentration immediately after the early Miocene unconformity [~20 million years ago (Ma)], concurrent with a shift in detrital zircon ages, indicating a major provenance shift. We identify bimodal volcanism of the Columbia River Basalt Group as the dominant Miocene source, requiring that the early Miocene Continental Divide lay ~400 kilometers west of its present position. This reorganization, driven by Yellowstone plume–induced uplift, persisted until at least 10 Ma. Our study provides geologically based, temporal, and spatial constraints on dynamic topography previously only estimated from modeling.

## Teaser

The sedimentary record reveals how Yellowstone’s rise transformed the landscape and displaced the Continental Divide.

## INTRODUCTION

The northern Rocky Mountains experienced a transition in tectonic regime during the Cenozoic (*1*). Sevier-Laramide compression ceased in the early Eocene (*2*) and was superseded by orogenic collapse with low-angle detachment faulting and the formation of metamorphic core complexes during the late Eocene–early Miocene (*3*), followed by the onset of high-angle normal faulting during Basin and Range extension (*4*, *5*) and the rise of the Yellowstone plume in the early Miocene (*6*, *7*). This transition not only transformed the landscape but also influenced faunal biogeography by facilitating species diversification through habitat fragmentation (*8*–*11*). While Oligo-Miocene sedimentary exposures are relatively sparse in western North America, the northern Rocky Mountains preserve a moderately thick and relatively well-exposed record within a series of intermontane basins (*12*). These basins captured the response of regional topography to tectonic and geodynamic processes, thereby providing insights into basin development, sediment dispersal patterns, and drainage evolution. As this region contains the modern North American Continental Divide, separating the watersheds draining into the Pacific Ocean from those draining into the Atlantic and Arctic oceans, it is particularly well suited for studying drainage migration and its relation to regional uplift.

Traditionally, drainage evolution has been inferred from provenance studies that analyze clast compositions and detrital zircon data [e.g., (*13*)]. However, rock magnetic studies of sedimentary sections provide a time and cost effective, high-resolution alternative by capturing variations in rock magnetic mineralogy through time, which may signal changes in sediment source. Magnetic methods allowed Gerritsen *et al.* (*14*) to identify an abrupt magneto-mineralogical change in a sedimentary section located at the Continental Divide near the Idaho-Montana border. This shift coincided with the formation of the regional early Miocene unconformity (EMU), which they precisely dated using magnetostratigraphy. Building on these initial findings, we present a comprehensive rock magnetic study of seven additional Oligo–Miocene stratigraphic sections in the northern Rocky Mountains. By integrating our high-resolution rock magnetic dataset with published detrital zircon records, we constrain the timing and spatial patterns of provenance changes associated with the EMU. This synthesis provides insights into regional uplift patterns, drainage divide migration, and shifts in sediment source in response to Cenozoic tectonics and mantle dynamics.

### Geologic setting

**Fig. 1.**
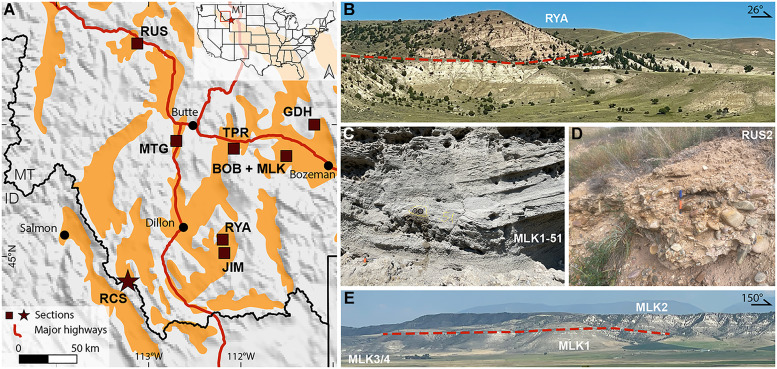
Map and field photographs of the study area in southwestern Montana (MT). (**A**) Map showing the basins containing Cenozoic sediments in orange (*12*) and shaded relief topography (*81*); brown squares indicate the locations of the seven sections sampled for this study and the brown star shows the Railroad Canyon section (RCS) along the Idaho (ID) border from Gerritsen *et al.* (*14*). The red star in the inset denotes the present position of the Yellowstone hotspot. Field photographs of (**B**) the RYA section, (**C**) fluvial sedimentary structures in the Sixmile Creek Formation in the MLK section, (**D**) conglomerate capping the EMU (early Miocene unconformity) in the RUS section, and (**E**) the MLK section. Red dashed lines indicate the interpreted positions of the EMU.

The Rocky Mountains constitute the largest mountain range in North America, stretching from New Mexico (USA) to British Columbia (Canada). They formed during the Sevier-Laramide Orogeny in the Late Cretaceous–Paleocene (*2*, *15*). Near the Montana-Idaho segment of the Rocky Mountains (Fig. 1A), herein referred to as the northern Rocky Mountains, the main constituent of the orogenic belt is the Precambrian Belt Supergroup (*16*), yet Paleozoic and Mesozoic sedimentary rocks (*17*) and Archean quartzofeldspathic gneiss (*18*) are also exposed. During the Late Cretaceous, the region was intruded by large magmatic bodies, such as the Pioneer, Boulder, and Idaho batholiths (*19*). Postorogenic extension began in the Eocene, forming the Bitterroot and Anaconda metamorphic core complexes and triggering volcanism, such as the Dillon, Challis, and Absaroka volcanic fields (*20*). Volcanism in southwestern Montana was relatively subdued in the early Miocene, with only limited eruptions (e.g., the Dillon volcanics near Dillon in Fig. 1A) (*21*–*23*).

Our study area in southwestern Montana is characterized by a series of north to south (N-S)–trending mountains separated by intermontane valleys (Fig. 1A). Details surrounding basin formation during the Eocene–early Miocene remain debated because of complications from subsequent Basin and Range extension. Hypotheses for basin development include: (i) formation of grabens and half-grabens through the reactivation of Sevier thrust faults (*3*); (ii) generation of grabens and half-grabens within a N-S–oriented rift zone located within the Sevier thrust belt, accompanied by an alluvial plain on the eastern rift shoulder (*24*); (iii) creation of a singular, expansive basin flanked by Eocene volcanic fields (*21*); and (iv) establishment of a network of fluvially incised valleys during the final stages of Sevier-Laramide orogenesis (*13*). Subsequent Basin and Range extension continued to create accommodation space during the Neogene, creating a complex network of rectilinear basins (*5*, *25*, *26*).

**Fig. 2.**
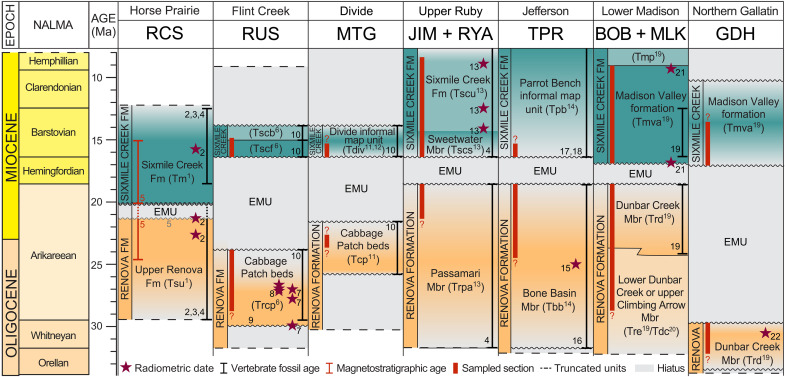
Correlation of the sections. Figure ordered from west to east [after figure 1 in Vuke *et al*. (*12*), reused with permission from S. Vuke and MBMG]. North American Land Mammal Ages (NALMA) from Barnosky *et al.* (*82*). Numbers in superscript, adjacent to stars, next to vertebrate fossil ages, and above unconformities in the figure artwork refer to the following references: ^1^M’Gonigle (*83*) ^2^Harris *et al.* (*84*) ^3^Barnosky *et al.* (*85*) ^4^Fields *et al.* (*28*) ^5^Gerritsen *et al.* (*14*) ^6^Portner and Hendrix (*65*) ^7^Calede (*86*) ^8^Portner *et al.* (*34*) ^9^Mosolf and Vuke (*87*) ^10^Calede and Rasmussen (*88*) ^11^Elliott and McDonald (*38*) ^12^Vuke (*89*) ^13^Brennan *et al.* (*90*) ^14^Vuke *et al.* (*91*) ^15^Link *et al.* (*92*) ^16^Axelrod (*93*) ^17^Kuenzi and Fields (*27*) ^18^Wang *et al.* (*94*) ^19^Vuke (*75*) ^20^Vuke (*76*) ^21^Montejo *et al.* (*95*) ^22^Hughes (*77*). Dotted lines in the RCS represent the possible extent of the EMU (≤1.5 Myr). Color gradients and question marks indicate age uncertainties.

Cenozoic sediments in these basins constitute the Bozeman Group (*12*). The Bozeman Group consists of two lithologically distinct depositional sequences, containing dissimilar fossil assemblages (*27*). The lower sequence, the Renova Formation, is generally characterized by fine-grained fluvial and lacustrine strata incorporating a large volume of volcanic ash, with conglomerate as a relatively minor component (*27*, *28*). In this study, we focus on the upper Renova Formation, of which fossils correspond to the Whitneyan-Arikareean stages of the North American land mammal age (NALMA) (Fig. 2 and references therein). The upper sequence, the Sixmile Creek Formation, predominantly consists of coarse-grained rocks, with increasing conglomerate layers toward the top (*27*, *28*). It contains Hemingfordian-Hemphillian NALMA fossils (Fig. 2 and references therein). Specific lithologic features of the Renova Formation may help distinguish it from the Sixmile Creek Formation: mollusk and insect fossils, paper shale, diatomaceous and coal beds, etc. (*12*), as well as rock color [e.g., Fig. 1B and Gerritsen *et al.* (*14*)]. However, these features are often missing, and the lithology of both formations is variable across the study area, making it sometimes challenging to distinguish the two.

The two formations are separated by the EMU, which has been identified in intermontane basins across southwestern Montana (*7*, *12*, *28*–*30*). Regionally, the EMU is categorized as an erosional and/or angular unconformity (*7*, *12*) or as a correlative conformity (*31*). The age and duration of the EMU vary between basins (Fig. 2). At the Railroad Canyon section (RCS) near the Idaho-Montana border, Gerritsen *et al.* (*14*) placed the end of the EMU at 20.1 million years ago (Ma) with a duration up to 1.5 Myr based on magnetostratigraphy in combination with zircon U-Pb ages.

We collected samples at the boundary between the Missouri and Columbia River drainage systems at the present-day Continental Divide. Paleocurrent analyses of the Renova [summarized by Vuke (*12*)] and Sixmile Creek [e.g., (*32*)] formations indicate northeast (NE)-flowing drainage systems similar to today. Clast compositions suggest that Renova Formation provenance was locally derived but may include Idaho sources (*13*, *33*). Sixmile Creek provenance was generally locally derived [e.g., (*22*, *23*, *34*)], but some clasts have affinity with sources from Idaho, Nevada, or Utah (*6*, *35*, *36*). A regional synthesis of late Miocene–Pliocene provenance data indicates a marked drainage reorganization linked to thermally induced topography of the Yellowstone plume tail (*37*). However, the effect of dynamic topography generated during the Oligo-Miocene rise of the Yellowstone plume head on drainage evolution remains unexplored. Using rock magnetic analyses in combination with published provenance data, our study provides a detailed analysis of the transition from the Oligocene Renova Formation to the early Miocene Sixmile Creek Formation in southwestern Montana.

## RESULTS

**Fig. 3.**
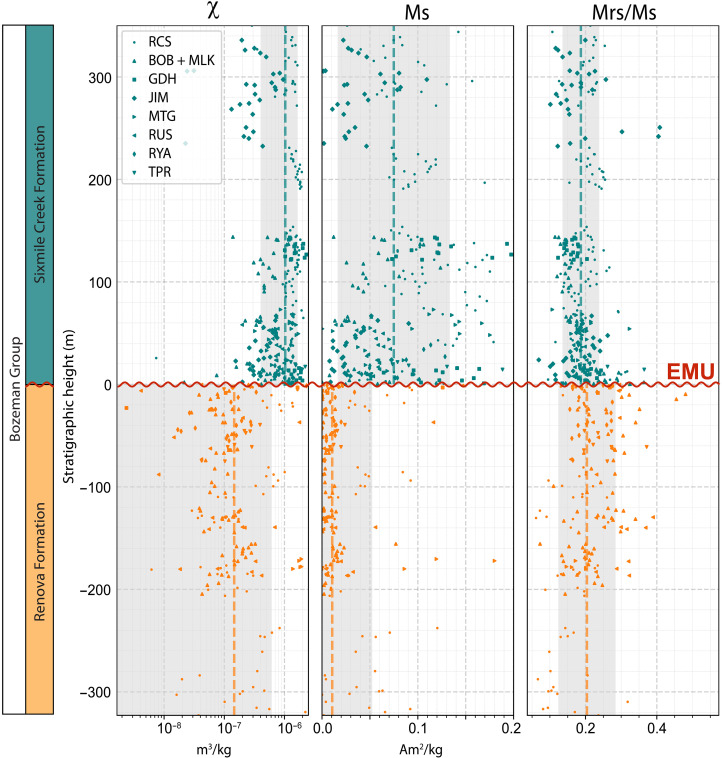
Rock magnetic properties of the eight sections. Sections aligned at the EMU (curvy red line) positioned at 0 m. From left to right: magnetic susceptibility (χ), Ms (saturation magnetization), and Mrs (saturation remanent magnetization) divided by Ms. Orange and teal points represent samples taken from the Renova Formation (*N* = 123) and Sixmile Creek Formation (*N* = 175), respectively. Dashed orange and teal lines indicate median values; shaded regions, 1σ uncertainty.

The stratigraphic position of the EMU coincides with a distinct change in rock magnetic properties between the Renova and Sixmile Creek formations (Fig. 3, figs. S1 to S8, and data S2). The magnetic mineral concentration, indicated by magnetic susceptibility (χ) and Ms (saturation magnetization), sharply increases immediately above the EMU in all sections (figs. S1 and S3 to S7) except in the MTG section (fig. S2), where the Renova Formation displays anomalously high magnetic mineral concentrations (Fig. 3). The median χ and Ms are 1.46 ± 4.64 × 10^−7^ m^3^/kg and 1.07 ± 4.17 × 10^−2^ Am^2^/kg below the unconformity and 1.03 ± 0.62 × 10^−6^ m^3^/kg and 7.49 ± 5.86 × 10^−2^ Am^2^/kg above, respectively. Magnetic grain size indicators, saturation remanent magnetization (Mrs) divided by Ms (Mrs/Ms; Fig. 3) and coercive force (Bc; fig. S8), have slightly lower medians in the Sixmile Creek Formation (0.19 ± 0.05 and 10.30 ± 3.13 mT) but still fall within the uncertainty of those for the Renova Formation (0.20 ± 0.08 and 13.60 ± 6.25 mT). The median hysteresis shape parameter (σ_hys_) is positive in the Renova Formation (0.17 ± 0.29), which reflects the presence of wasp-waisted loops, indicative of a mixture of magnetic minerals with contrasting coercivities (fig. S8). Moreover, the Renova Formation has lower S-ratios on average (0.93 ± 0.10) compared to the Sixmile Creek Formation (0.97 ± 0.03), indicating a greater abundance of high coercivity minerals such as (titano)hematite (fig. S8; see Materials and Methods). Overall, rock magnetic properties are more uniform in the Sixmile Creek Formation, dominated by low-Ti titanomagnetite (fig. S9), whereas the Renova Formation displays more heterogeneity, with lower magnetic mineral concentrations that are still dominated by low-Ti titanomagnetite but slightly mixed with (titano)hematite.

**Fig. 4.**
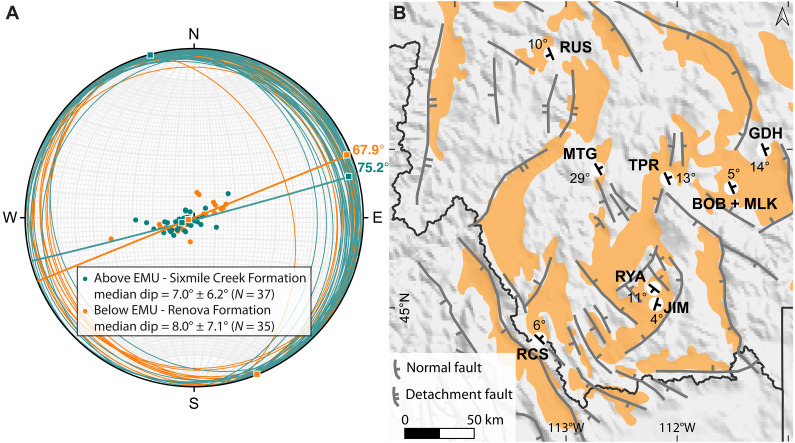
Summary of measured bedding planes. (**A**) Lower hemisphere stereographic projection of the measured bedding planes and poles to bedding. Renova Formation in orange and Sixmile Creek Formation in teal; maximum extension directions are 67.9° ± 8.9° and 75.2° ± 16.3°, respectively. (**B**) Map showing the average measured bedding planes at each section. Basins containing Cenozoic sediments are in orange (*12*); major extensional faults are in gray [after figure 1 in Janecke (*5*)]. N, north; W, west; S, south; E, east.

The measured bedding planes define similar northwest (NW)-oriented tilt axes, with extension directions oriented 75.2° ± 16.3° for the Sixmile Creek Formation and 67.9° ± 8.9° for the Renova Formation (Fig. 4A and data S2). The median dip of both formations is low: 7.0° ± 6.2° (*N* = 37) and 8.0° ± 7.1° (*N* = 35) for the Sixmile Creek and Renova formations, respectively. At first glance, the bedding planes suggest a continuous tectonic regime above and below the EMU; however, most bedding planes dip west for the Renova Formation, while those for the Sixmile Creek Formation dip east. For example, in the Ruby Basin, the Renova Formation at RYA dips west (Fig. 4B and fig. S4), while the lower Sixmile Creek Formation at JIM dips east (Fig. 4B and fig. S3). Although our dataset is limited, this contrast might indicate differences in fault activation between the two formations, with NE-dipping listric or normal faults more active in the Oligocene and SW-dipping faults active in the Miocene. Further work should be carried out to resolve this.

## DISCUSSION

Our rock magnetic study reveals an abrupt increase in magnetic mineral concentration (χ and Ms) immediately after the EMU across southwestern Montana (Fig. 3). Given the U-Pb ages in the sections and the precise age constraints of the EMU in the RCS (*14*), we find that the enrichment in magnetic minerals persisted from 20.1 Ma until at least 10 Ma (Fig. 2). As stated earlier, identifying the EMU is routine in some sections (e.g., a lithologic color change; Fig. 1B and figs. S4 and S7); whereas it is challenging in others (fig. S5) (*12*). However, the transition is clearly detectable with magnetic data, which are inexpensive and time efficient to obtain.

The MTG section is the sole exception to this trend (fig. S2), where the Renova Formation exhibits anomalously high magnetic mineral concentrations (Fig. 3). Given that the formations are difficult to distinguish at this location (*38*), it is possible that the lower part of the MTG section belongs to the Sixmile Creek Formation rather than the Renova Formation. In addition, the presence of faults crosscutting the section complicates the stratigraphy. Moreover, a limited number of samples were taken from the Renova Formation (five samples from 10 m of section). We regard this section as an outlier and interpret an increase in magnetite concentration directly above the EMU as valid over the entire study area.

### Climatic and pedogenic influences

The Renova Formation appears to contain more (titano)hematite compared to the overlying Sixmile Creek Formation (fig. S9), which could indicate that climate was different below and above the EMU. Oxidation of magnetite to the much less magnetic hematite could explain lower χ and Ms values, lower S-ratios, and positive σ_hys_ in the Renova Formation (Fig. 3 and fig. S8). However, if this were the case, we would expect different paleomagnetic polarities between the magnetite and hematite unblocking spectra (~500° to 580°C and ~600° to 680°C, respectively). In 123 thermally demagnetized samples from the RCS, including 41 from the Renova Formation, none displayed polarity or directional differences with distinct magnetization components segregated by unblocking temperature (*14*). Furthermore, magnetic minerology remains relatively constant for ~10 Myr in both the Renova and Sixmile Creek formations (Figs. 2 and 3 and fig. S8), so the differences are not transitory signals. Climatic proxies reveal no clear shift at the EMU (*15*, *39*–*41*), meaning that the oxidation state of the magnetic minerals is unlikely to be attributed to long-term weather conditions (temperature, humidity, etc.) or pedogenic processes.

### Compilation of detrital zircon data

Having ruled out the influence of climatic or pedogenic processes, the most plausible explanation for the shift in magnetic mineralogy is a change in sediment source following the EMU. The provenance must have changed from a magnetite-poor (e.g., sedimentary rocks) to a magnetite-rich source (e.g., crystalline basement or volcanic rocks). A common method to determine provenance is to study detrital zircon age spectra, which provide information on the age of the source rocks of the sediments. By combining our detailed rock magnetic study with detrital zircon data, we attempted to identify possible source areas. We note that the changes in magnetic and detrital zircon signals are not necessarily coupled; basaltic volcanism is rich in magnetite but poor in zircon, whereas silicic volcanism is poor in magnetite but richer in zircon. This decoupling means that an influx of basaltic material could enhance magnetite concentration independent of the zircon age spectrum.

**Fig. 5.**
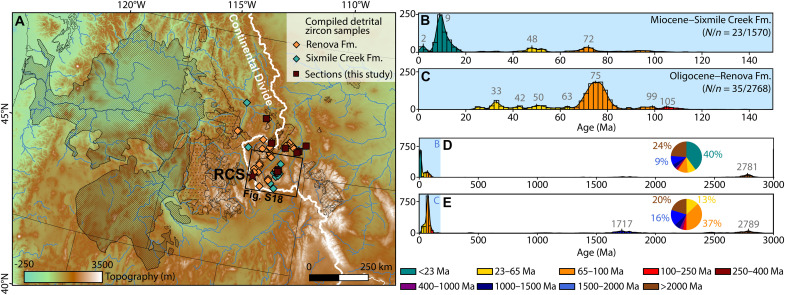
Detrital zircon data compilation. (**A**) Topographic map of the northwestern United States (*81*), showing the present-day Continental Divide (white line), our sections, and the locations sampled for detrital zircon analysis. Legend for shaded regions in Fig. 6. (**B** to **E**) Composite detrital zircon age histograms for the Renova and Sixmile Creek formations with the <150 Ma spectrum in (B) and (C) and full spectrum in (D) and (E) (*35*, *37*, *43*, *44*, *53*, *92*, *96*); *N*/*n* = number of samples/number of zircon ages.

We compiled detrital zircon age data that characterize the Renova (*N*/*n* = 35/2768; *N* = number of samples, *n* = number of zircon ages) and Sixmile Creek (*N*/*n* = 23/1570) formations from southwestern Montana (Fig. 5 and data S3). By creating composite age spectra for the two formations, we spatially and temporally average the data, thereby highlighting the common source contributors while reducing local variants. During both Renova and Sixmile Creek times, fluvial interconnection between different basins allowed common sources to be transported across basins (*6*, *12*, *42*), while nearby topographic highs contributed proximal sources (*13*, *43*). The use of composite detrital zircon data for each formation aligns with the distinctly different magnetic properties between the two formations yet relative consistency within each formation.

The composite age spectrum of the Renova Formation is dominated by a Late Cretaceous signal (mostly 85 to 65 Ma), constituting 37% of all ages (Fig. 5, C and E). The nearby Late Cretaceous Boulder and Pioneer batholiths, and potentially the Idaho Batholith, likely sourced these zircons (*43*, *44*). A pre-1500 Ma signal comprises 36% of all zircons (Fig. 5E); peaks occurring at ~1.7 and ~2.8 billion years ago (Ga) are consistent with nearby Mesoproterozoic Belt Supergroup (*43*) and Archean crystalline basement (*18*), respectively. Paleogene zircons (13%; 23 to 65 Ma; Fig. 5, C and E) most likely originate from the nearby Dillon, Absaroka, and Challis volcanic fields [42 to 55 Ma; (*45*)] and ash sourced from the Cascade Arc [≤43.2 Ma; (*46*)]. The youngest zircon ages within the Renova Formation reach up to 22.7 Ma (data S3), which agrees with its depositional age (Fig. 2). Minor contributions consist of zircons with ages of 100 to 250 Ma (4%), 250 to 400 Ma (1%), 400 to 1000 Ma (3%), and 1000 to 1500 Ma (6%).

The composite age spectrum of the Sixmile Creek Formation is considerably different from that of the Renova Formation, with a dominant Miocene signal (<23 Ma, peak at 9 Ma) that encompasses 40% of all zircon ages (Fig. 5, B and D). This component replaces the Late Cretaceous dominance of the Renova Formation, which comprises only 11% in the Sixmile Creek Formation. The contribution of Paleogene zircons remains similar at 9% (Fig. 5D), but the younger 33 Ma peak is absent, further indicating that this was likely supplied by ash from the Cascade Arc to the Renova Formation. Meanwhile, the pre-1500 Ma contribution remains high in the Sixmile Creek Formation, comprising 33% and containing a peak at 2.8 Ga (Fig. 5D). Smaller contributions of zircons with ages of 100 to 250 Ma (1%), 250 to 400 Ma (1%), 400 to 1000 Ma (2%), and 1000 to 1500 Ma (4%) are present.

### Sediment source identification

**Fig. 6.**
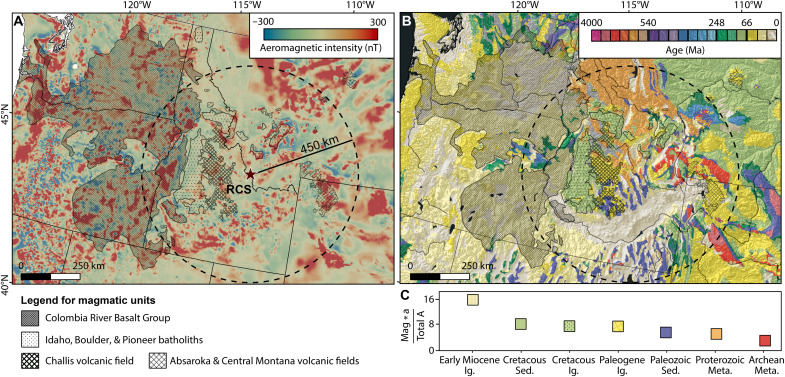
Magnetic contribution of major geologic units. (**A**) Aeromagnetic (*97*) and (**B**) geologic (*17*) map of the northwestern United States. The 450-km radius, dashed black circle centered on the RCS, defines the area considered for the magnetite source for the Sixmile Creek Formation. (**C**) The estimated magnetic contribution of each major geologic unit (see text for details). Shaded regions indicate the surface exposure of large magmatic units.

Pre–150 Ma detrital zircons found in both formations could be recycled from older sediments (*43*). However, the presence of zircons with Precambrian ages is consistent with clast count analyses, which indicate that basement rocks were already exposed during Renova deposition (*13*). Some mafic dikes within the basement (*18*) could potentially be a magnetite-rich source, as seen on the aeromagnetic map (Fig. 6A); however, they should have contributed to both formations equally. Furthermore, the exposure of basement rocks would not explain a simultaneous provenance shift in all basins. Therefore, it appears unlikely that the basement rocks are the source of magnetite above the EMU.

If the magnetic and detrital zircon signals are coupled, i.e., if they come from the same rock unit, it becomes difficult to find suitable candidates of Miocene age near our sections. The Dillon volcanic field is the closest volcanic center that has a Miocene eruption phase (*21*–*23*), making it a potential candidate. Today, it has a very limited extent, but it was possibly more widespread than we can infer from its current exposure. However, the Dillon volcanics erupted mostly during the Eocene (*21*, *22*), so they should have contributed to the Renova Formation as well. Moreover, when taking the paleo-drainage into account (*42*), the Dillon volcanic rocks lay downstream of some of our sections (e.g., the RCS; fig. S10); hence, we think that Dillon volcanism cannot represent a substantial source of magnetite in the Sixmile Creek Formation.

**Fig. 7.**
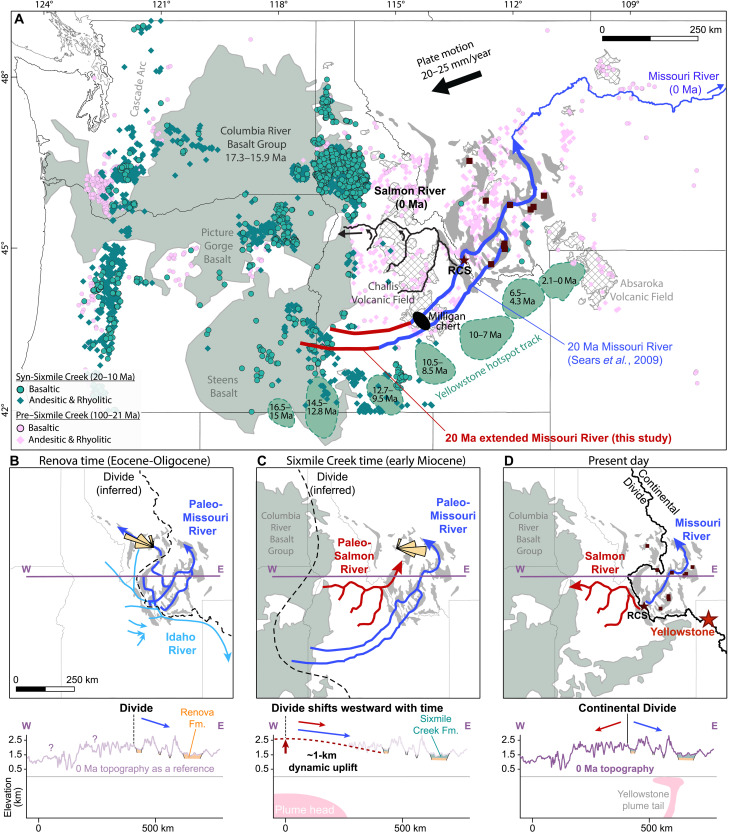
Drainage evolution of the northwestern United States. (**A**) Location of 100 to 10 Ma magmatism in the northwestern US compiled from the North American Volcanic and Intrusive Rock Database (NAVDAT) dataset (*98*) and Schwartz *et al.* (*43*). Bold red lines indicate the proposed extended Miocene Paleo-Missouri River. (**B** to **D**) Detailed evolution of the paleodrainage. (B) Eocene Idaho River in light blue (*33*, *62*), Eocene-Oligocene Paleo-Missouri River in dark blue [compiled by Vuke (*12*)]; rose diagram (*N* = 21) from Portner and Hendrix (*65*). (C) Miocene Paleo-Missouri River in dark blue (*6*), Paleo-Salmon River in red (*64*); rose diagram (*N* = 13) from Portner and Hendrix (*65*). (D) Present-day drainage situation with the projected position of the Yellowstone plume tail. Brown squares indicate sampling localities (Fig. 1); star indicates the RCS.

Although local volcanism during the Oligo-Miocene was limited, distal volcanism in the Cascade Arc was abundant during this time and could have supplied large volumes of ash, and thus magnetite, to southwestern Montana (Fig. 7A). Cascade products contributed to the Renova Formation during the Oligocene, as evidenced by its high ash content [e.g., (*28*)], as corroborated by regional volcanic records (fig. S11) (*47*). However, deposition of the Sixmile Creek Formation coincided with a pronounced decline in volcanic productivity during the early Miocene, with silicic output dropping more than an order of magnitude (*46*, *48*, *49*), whereas we observe a marked increase in magnetite concentration (fig. S11). Volcanism during this time was dominated by the Yellowstone plume that emplaced the voluminous basaltic lavas of the Columbia River Basalt Group (CRBG) but little explosive ash (Fig. 7A). Cascade-derived air fall deposits within the CRBG are thin and locally restricted (*48*, *49*); the limited Yellowstone-related explosive volcanism that did occur was relatively iron poor, especially after 15 Ma (fig. S11) (*50*, *51*). Although fluvially reworked tephra beds in the Sixmile Creek Formation can locally exceed 30 m, individual ash fall deposits interbedded within alluvial fans were likely no thicker than ~10 cm in our study area (*52*). Inherited zircons within sampled tuffs reinforce this picture of ash reworking (*53*). The fluvial dominance of the Sixmile Creek Formation is further reflected in the lithology, where air fall tuff facies are described only as minor constituents (*28*, *32*, *34*, *54*), consistent with our field observations of cross-bedding, channels, and gravel lenses (Fig. 1C and fig. S7). Collectively, these lines of evidence suggest that ash fall alone is insufficient to account for the observed magnetite enrichment, which must have been supplied primarily through fluvial transport.

We used aeromagnetic anomaly and geologic maps within a radius of 450 km around the RCS to evaluate the fluvial delivery of magnetite (Fig. 6). The factors that guided our choice of potential candidates were the intensity of the aeromagnetic anomalies (Fig. 6A) and the exposed surface area of each geologic unit older than 16 Ma (Fig. 6B). For a source to provide input to the large area of the Sixmile Creek Formation for a prolonged time (>10 Ma), there must be a relatively large exposed surface area. Figure 6C presents the total magnetic contribution of each major geologic unit by taking its average aeromagnetic intensity (Mag) and exposed surface area (a) normalized by the total explored area (total A). This analysis clearly shows that early Miocene igneous rocks of the CRBG are the greatest magnetic source, with more than twice the total magnetic contribution compared to other units. Cretaceous igneous rocks of the Idaho, Boulder, and Pioneer batholiths have relatively low magnetic contributions. These batholiths are primarily composed of two-mica granites, granodiorites, or quartz monzonites (*55*, *56*), which lack magnetite. Being a main source for the Renova Formation (37%; Fig. 5E), this explains the weak magnetic signal in this formation. Although some of the sedimentary rocks have relatively high magnetic intensities (e.g., Cretaceous Sed. in Fig. 6C), the sources likely come from underlying units (*57*).

The Yellowstone plume head eruption produced >98% of the CRBG volume between 16.7 and 15.9 Ma (*58*). These basalt flows are rich in low-Ti titanomagnetite (*59*), identical to what we observe in the Sixmile Creek Formation. Synchronous rhyolitic centers could have provided airborne ash (Fig. 7A) (*60*) or fluvially transported zircons to our study area. We note, however, that this ash is low in iron, particularly after 15 Ma (*50*, *51*), while magnetite concentrations in our sections are consistently high between 20 and 10 Ma (fig. S11). In other words, the bimodal volcanism of the CRBG could explain the signals in the Sixmile Creek Formation by providing Miocene zircons from the rhyolitic rocks together with magnetite from the basaltic rocks. The location of the CRGB is also consistent with paleocurrent data that suggest a source area to the southwest (fig. S10). Adding the CRBG as a source, however, requires the Continental Divide to have been located ~400 km west of its present-day position (Fig. 5A), as discussed in the next section.

If the CRGB alone contributed the magnetite, a problem arises to explain why magnetite concentration increased already by ~20 Ma (the age of the EMU in the RCS; Fig. 2), about 3 Myr before the oldest known age for the CRGB [17.3 Ma; (*61*)]. Perhaps older CRGB flows do exist but are covered by younger ones or the older units that did exist were subsequently eroded. The presence of a 20.8 ± 0.6 Ma zircon from the Sixmile Creek Formation might hint at the existence of older flows (*53*). Alternatively, some magnetite could have been shed from Paleogene basalts of the Challis volcanic field, which are located upstream of our study area (Fig. 7A). In this scenario, contribution from the Challis basalts again requires a westward shift of the drainage divide commensurate with the EMU. Other far-field sources to the southeast of our study area, such as the Absaroka volcanic field, are unlikely since they are located downstream (Fig. 7A) (*6*, *42*).

In short, the early Miocene CRBG provides the most straightforward explanation for the source of the magnetite and the Miocene detrital zircon age peak in the Sixmile Creek Formation, perhaps with some contribution of magnetite from the Challis basalts. This requires the Continental Divide to have shifted westward simultaneously with the formation of the EMU.

### Early Miocene shift of the Continental Divide

Our studied sections are distributed across the present-day Continental Divide (Fig. 7D). For example, the RUS section lies within the Columbia River watershed, the RCS and MTG sections lie on the Continental Divide, and the other sections are situated within the Missouri River watershed. Therefore, the current Continental Divide separates the RUS section from our other sections. Given the similar magnetic characteristics observed in all the sections (Fig. 3 and figs. S8 and S9), an interconnected fluviolacustrine system must have existed, linking them into a single, coherent drainage system during the Renova and Sixmile Creek periods. This suggests that the present-day Continental Divide was established after 10 Ma, the uppermost limit of our dataset.

In general, paleocurrent measurements from the Renova Formation [Fig. 7B; compiled by Vuke (*12*)] and the Sixmile Creek Formation [Fig. 7C; compiled by Schwartz and Schwartz (*13*)] indicate NE-directed flow, similar to the present-day Missouri River (Fig. 7D). The Renova provenance is dominated by weakly magnetic material with peak detrital zircon ages at 75 Ma. While we cannot rule out far-field sources like the Idaho Batholith, a closer source from the Boulder and Pioneer batholiths in combination with recycled zircons can sufficiently explain our observations (*13*, *43*). An Eocene river has been proposed to exist in the western part of our study area (Fig. 7B). This river is thought to have flowed from Idaho, through Montana, and into Wyoming, based on similarities in lead isotopes between the Challis volcanic rocks and coeval sediments in Wyoming (*33*, *62*). Given this regional paleogeographic context, a local source from the Boulder and Pioneer batholiths would be more compatible with regional paleogeographic reconstructions. The Eocene-Oligocene paleogeography in southwestern Montana thus appears to have been similar to the present-day landscape (*13*).

We propose that the early Miocene drainage system of southwestern Montana included the CRBG, which lies ~400 km west of the Continental Divide (Fig. 7C). Given that the present-day Continental Divide is broadly distributed over a wide area (cross section in Fig. 7D), slight adjustments (hundreds of meters) in local relief can highly affect the position of the divide. Sears and Ryan (*63*) previously suggested a far-field source for the Sixmile Creek Formation, identifying exotic chert clasts in the Ruby Basin (our JIM section) and linking them to the Milligan chert in Idaho. They proposed that the Miocene paleo-Missouri River headwaters extended ~200 km west of the present-day divide. Our findings indicate that the headwaters extended even farther west, incorporating the CRBG into the source area. A drainage connection between Idaho and southwestern Montana has also been speculated on the basis of the NE-oriented tributaries and the sharp 90° turn of the Salmon River (Fig. 7A). Anderson (*64*) proposed that the Salmon River once flowed along what is now the inverted course of the Carmen Creek, draining toward the present-day Missouri River (Fig. 7C). Anderson (*64*) further observed NE-oriented wind gaps—dry valleys or passes that once carried rivers—indicating paleo-drainage pathways from Idaho to southwestern Montana. Paleocurrent measurements obtained from imbrication in the conglomerate capping the EMU in the Flint Creek basin (*65*), within our RUS section (Fig. 1D), also indicate an opposite flow direction during the early Miocene (Fig. 7C).

All in all, while local sources undoubtedly contributed to our source region—such as alluvial fans sourced from adjacent mountains (*13*)—our findings demonstrate that a through-going drainage system with far-field sources during the early Miocene is essential to account for both the magnetic and detrital zircon signals. This challenges previous interpretations invoking predominantly local sources for the western Montana basins (*13*, *66*). Our data strongly support that notable changes occurred in the drainage network during the late Oligocene-Miocene.

### Controls on drainage reorganization

A shift in the tectonic regime, such as the onset of Basin and Range extension and/or dynamic uplift from the Yellowstone plume, can cause drainage reorganization. The influence of Basin and Range deformation is evident from our observations, with gently NE or SW dipping bedding planes (Fig. 4) aligned roughly parallel to the extension direction of 45°N measured by focal plane solutions (*67*, *68*), perpendicular to the major extensional faults in the region (Fig. 4B). These low dip angles are likely caused by high-angle normal faulting, as is common during Basin and Range extension (*5*, *69*). Our limited dataset shows that bedding planes of the Sixmile Creek Formation predominantly dip NE, suggesting that SW-dipping listric or normal faults were more active in the Miocene, while the opposite pattern prevailed in the Oligocene. Although Basin and Range deformation clearly influenced the region, the drainage evolution we propose, in which the present-day drainage configuration reverts to its Eocene-Oligocene state (Fig. 7, B to D), necessitates a long-wavelength and transient topographic pulse.

For the drainage divide to shift and incorporate the CRBG into the source area, the CRBG needed to be ~1 km higher than its present elevation (Fig. 7D). The horizontal and vertical scaling is consistent with current estimates of dynamic topography: elevation changes caused by lateral density variations and convective flow in the mantle. Dynamic topography from the Yellowstone plume head extended up to 1000 km horizontally from the plume center (*70*). A maximum vertical amplitude of ~1000 m is suggested for dynamic topography on the continents (*71*), which has been proposed as a driver of large-scale drainage reorganization (*6*, *7*). Moreover, analog modeling indicates that dynamic uplift precedes flood basalt eruptions by at least 3 Myr (*72*), in line with our findings that drainage shifts occurred around 20 Ma. After the plume head erupted as the CRBG, uplift was no longer dynamically supported and waned, causing the drainage divide to eventually return to its preuplift position. Our results indicate that the CRBG continued to supply sediments to southwestern Montana until at least 10 Ma (Figs. 2 and 3), implying that dynamic topography persisted for at least 10 Myr. Our results thus support a whole-mantle Yellowstone plume scenario, as suggested by the geodynamic model of Steinberger *et al.* (*73*). An alternative origin of the CRGB—driven by asthenosphere flow from the Pacific through slab tear (*74*)—is not preferred, since it would require dynamic uplift to be induced only after slab breakoff at 17 Ma, which is inconsistent with our observations. Our study therefore provides temporal and spatial constraints on dynamic topography previously only estimated from modeling.

In summary, our study reveals a pronounced increase in magnetic mineral concentration directly above the EMU across southwestern Montana, signaling a major shift in sediment provenance that is unrelated to climate change. Low magnetic concentrations combined with a predominance of 85 to 65 Ma zircon ages in the Renova Formation reflect sediment input primarily from Cretaceous batholiths, while the high magnetite content in the Sixmile Creek Formation points to the introduction of a new source during the early Miocene. The CRBG stands out as the primary candidate for this new source, as its bimodal volcanism provides both the magnetite and the Miocene-aged zircons observed in the sediments. This shift in provenance requires a substantial westward migration of the Continental Divide during the latest Oligocene to early Miocene, driven by dynamic uplift from the Yellowstone plume.

Our rock magnetic observations provide robust constraints on geodynamic model predictions, underscoring the importance of interdisciplinary approaches to linking mantle flow and surface processes. If the Renova drainage pattern was similar to the present-day, then the sediment source must have returned to a magnetite-poor lithology after 10 Ma. Future research should investigate late Neogene sedimentary sections to confirm such a shift by tracking declines in magnetic mineral concentration. In addition, further paleocurrent and provenance studies are needed to refine the timing and extent of the early Miocene drainage reorganization. Such studies will deepen our understanding of the complex interactions between mantle dynamics and surface processes in North America, revealing critical insights into how mantle processes shape continental-scale drainage systems.

## MATERIALS AND METHODS

We conducted a rock magnetic study above and below the EMU in the northern Rocky Mountains (Fig. 1). Samples were collected during a field campaign in 2023 that netted 561 samples from seven composite sections (figs. S1 to S7). Data from the RCS were described by Gerritsen *et al.* (*14*). Paleomagnetic cores were obtained using a battery-powered, water-cooled drill; poorly lithified strata were sampled with a handheld corer. We collected samples at roughly 2-m intervals throughout each section, obtaining two oriented cores per horizon (data S1), focusing on fine-grained materials, including silt and fine sandstones.

The average bedding attitude for each section (data S2) was used to project the Global Positioning System (GPS) position of each sample into a composite stratigraphic column per section (data S1). We could not determine the stratigraphic position of the BOB section with respect to the MLK section due to the large distance between them. According to Vuke (*75*), the lowest map unit of the MLK section correlates with the map unit we sampled in the BOB section (*76*); therefore, we placed the base of the BOB section 60 m below the MLK section.

The stratigraphic position of the EMU was determined differently for each section. The EMU was usually accompanied by lithologic changes, such as a change in color (RYA section; Fig. 1B and fig. S4), the presence of a conglomerate layer directly above the EMU (RUS section; Fig. 1D and fig. S1), or a distinct resistant unit of stacked calcic paleosols (MLK section; Fig. 1E and fig. S6). Satellite images helped identify the location of the EMU in the GDH section (fig. S7), where we projected the observed position of the EMU on a map taking the average bedding attitude of the section (dip direction = 62°N, dip = 14°). Our position of the EMU lies west of the EMU according to the geologic map (*75*) but is in good agreement with Hughes (*77*). For the MTG, JIM, and TPR sections, we relied on geologic maps to situate the stratigraphic position of the EMU (figs. S2, S3, and S5).

We measured the rock magnetic properties of bulk samples to assess variations in magnetic mineral composition and grain size. A Bartington MS2 meter characterized the low-field (465 Hz) χ. Backfield curves (which define the coercivity of remanence, Bcr) and hysteresis loops were determined with a LakeShore MicroMag 3900 vibrating sample magnetometer. HystLab software (*78*) helped interpret the hysteresis loops to obtain the Bc, Ms, Mrs, and σ_hys_ (*79*). S-ratios were obtained by first applying a 1 T saturation isothermal remanent magnetization (SIRM) in the sample’s +*y* axis, followed by a backfield IRM of −0.3 T in the −*y* axis: $S=\left[\left(-{\text{IRM}}_{-0.3T}/\text{SIRM}\right)+1\right]/2$ (*80*). The S-ratio is a proxy for the relative hematite to magnetite concentration, with a value of 1.0 indicative of magnetite and 0.5 for samples rich in high coercivity minerals (>300 mT) like hematite.

## Supplementary Materials

### The PDF file includes:

Figs. S1 to S11 Legends for data S1 to S3 References
