# Supplementary material for: Yellowstone plume drives drainage reorganization in the early Miocene
Source: Sci Adv. 2025 Nov 19;11(47):eadz4275. doi: 10.1126/sciadv.adz4275 (PMC12629190; doi:10.1126/sciadv.adz4275)
Supplement: Supplementary file 1 — Figs. S1 to S11 Legends for data S1 to S3 References [file sciadv.adz4275_sm.pdf]

Science Advances
AAAS

Supplementary Materials for

Yellowstone plume drives drainage reorganization in the early Miocene

Dieke Gerritsen *et al.*

Corresponding author: Dieke Gerritsen, h.gerritsen@lmu.de

Sci. Adv. **11**, eadz4275 (2025)
DOI: 10.1126/sciadv.adz4275

The PDF file includes:

Figs. S1 to S11
Legends for data S1 to S3
References

Other Supplementary Material for this manuscript includes the following:

Data S1 to S3

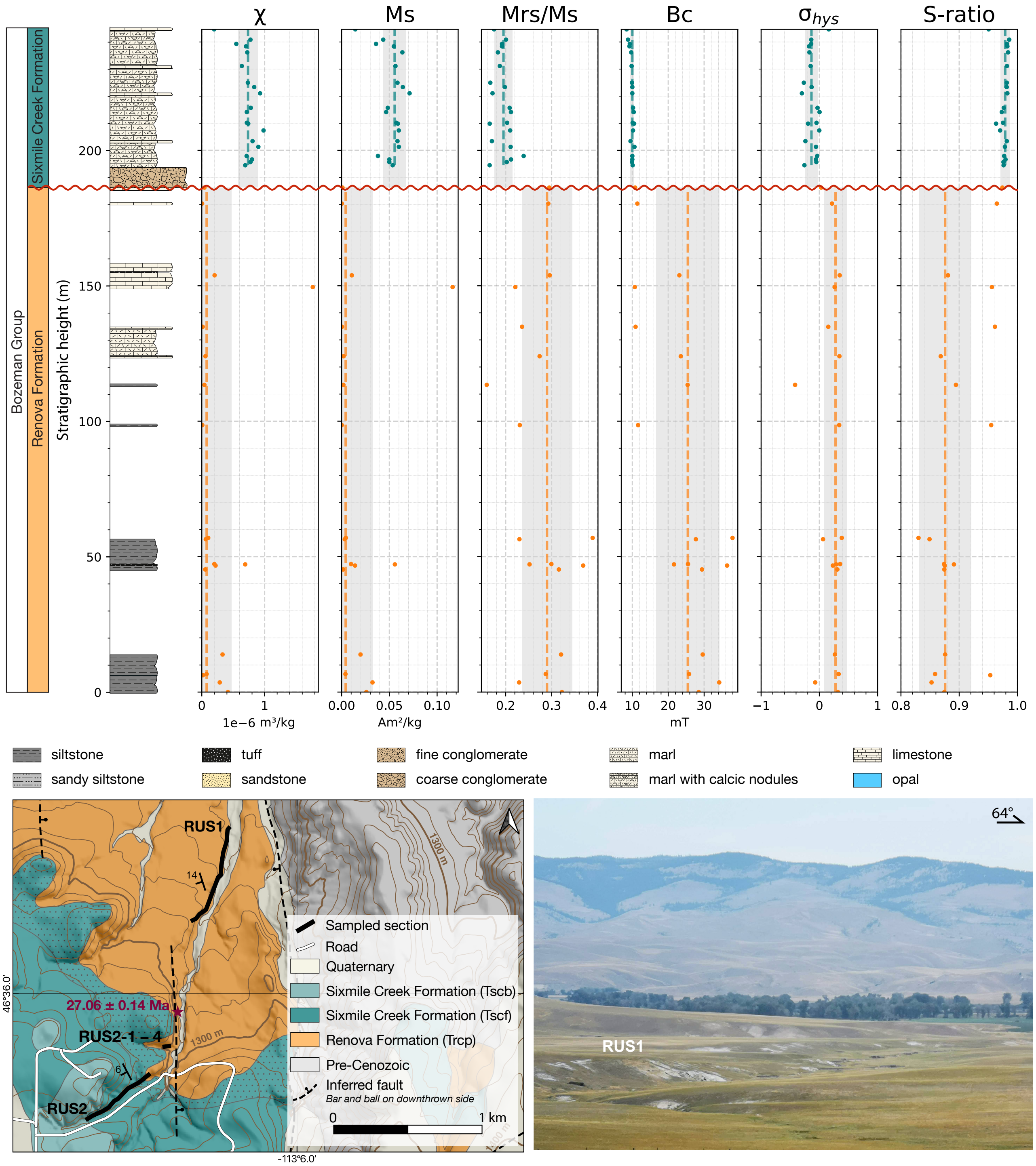

Fig. S1. Overview of the RUS section. Top: Simplified sedimentary log and rock magnetic properties. From left to right: magnetic susceptibility (χ), saturation magnetization (Ms), saturation remanent magnetization (Mrs) divided by Ms, coercive force (Bc), hysteresis shape parameter (σ_{hys}), and hematite to magnetite concentration (S-ratio). Orange and teal points represent samples taken from the Renova Formation (N = 19) and Sixmile Creek Formation (N = 20), respectively. Dashed orange and teal lines indicate their median values (gray shaded regions 1σ uncertainty). Curvy red line represents the early Miocene unconformity (EMU). Bottom left: Geologic map (*65*). The dotted unit on the geologic map represents a conglomerate at the base of the Sixmile Creek Formation, immediately above the EMU (Fig. 1D). Red star indicates location of U-Pb age with 2σ uncertainty (*34*). Bottom right: Field photograph.

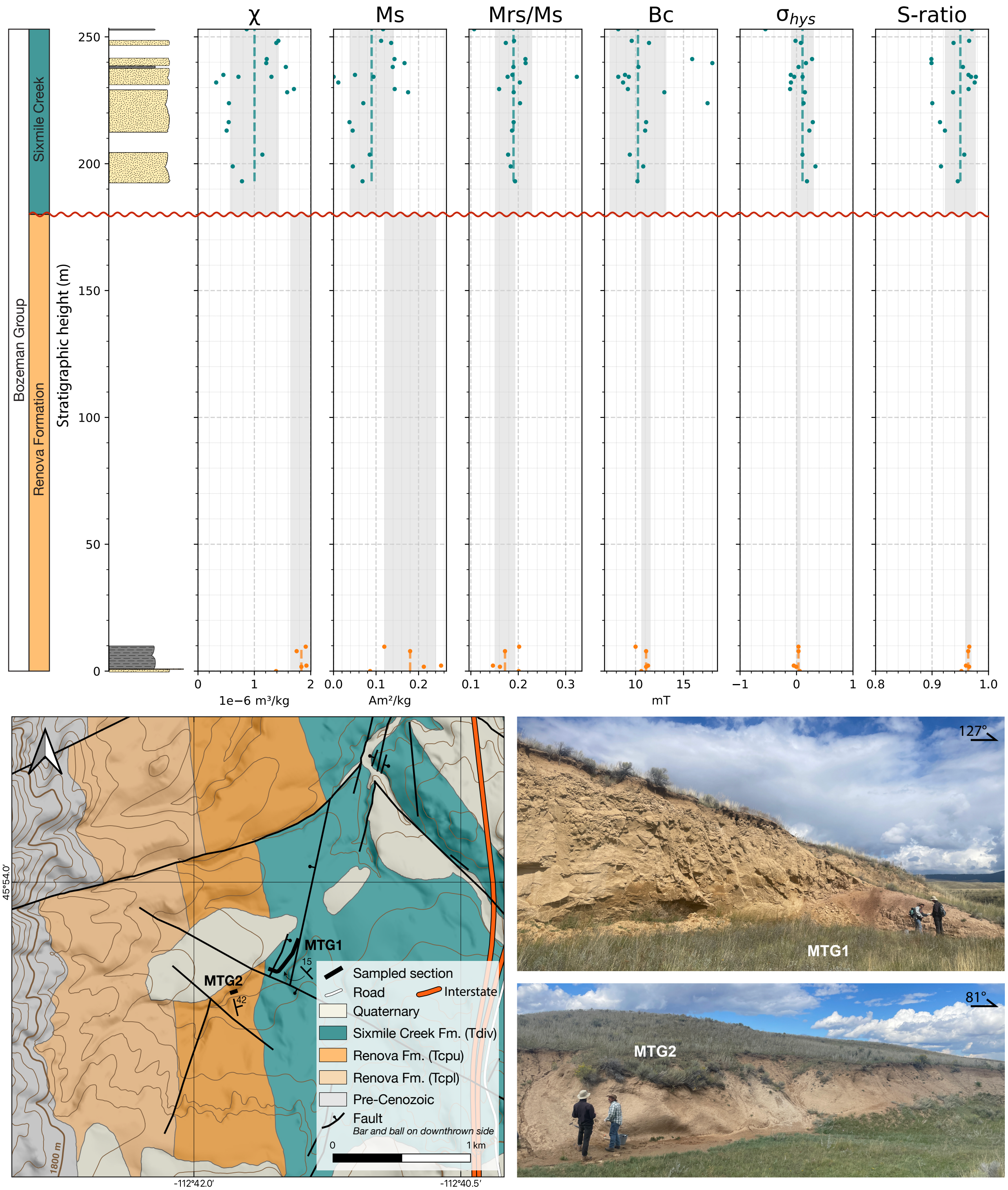

Fig. S2. Overview of the MTG section. Top: Simplified sedimentary log (legend in fig. S1) and rock magnetic properties. From left to right: magnetic susceptibility (χ), saturation magnetization (Ms), saturation remanent magnetization (Mrs) divided by Ms, coercive force (Bc), hysteresis shape parameter (σ_{hys}), and hematite to magnetite concentration (S-ratio). Dashed orange and teal points represent samples taken from the Renova Formation (N = 5) and Sixmile Creek Formation (N = 18), respectively. Orange and teal lines indicate their median values (gray shaded regions 1σ uncertainty). Curvy red line represents the early Miocene unconformity (EMU). Bottom left: Geologic map (*38*). Bottom right: Field photographs.

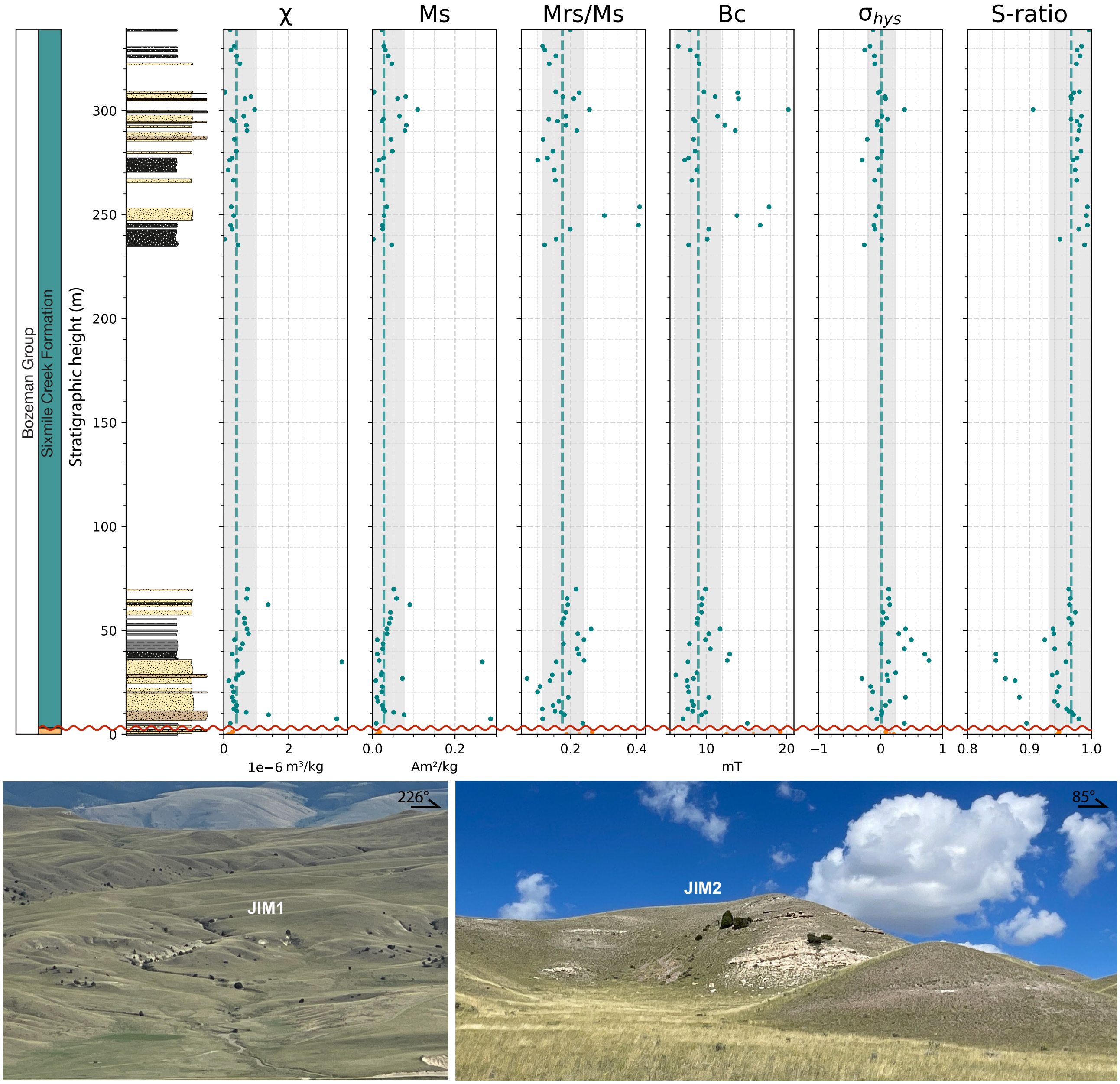

Fig. S3. Overview of the JIM section. Top: Simplified sedimentary log (legend in fig. S1) and rock magnetic properties. From left to right: magnetic susceptibility (χ), saturation magnetization (Ms), saturation remanent magnetization (Mrs) divided by Ms, coercive force (Bc), hysteresis shape parameter (σ_{hys}), and hematite to magnetite concentration (S-ratio). Orange and teal points represent samples taken from the Renova Formation (N = 2) and Sixmile Creek Formation (N = 56), respectively. Dashed orange and teal lines indicate their median values (gray shaded regions 1σ uncertainty). Curvy red line represents the early Miocene unconformity (EMU). Bottom: Field photographs.

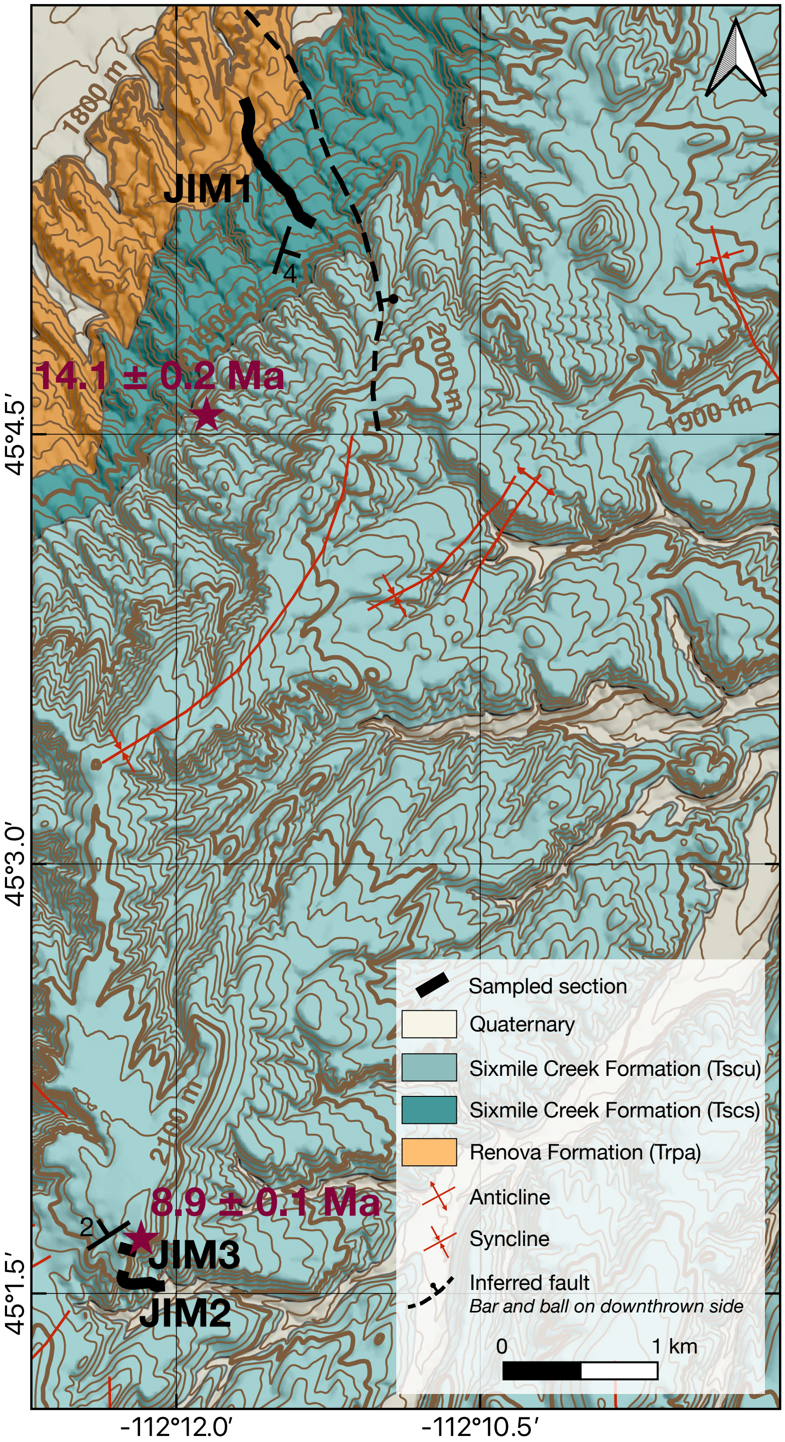

Fig. S3 (cont.). Geologic map, with red stars indicating locations of U-Pb ages with 2σ uncertainties (*90*).

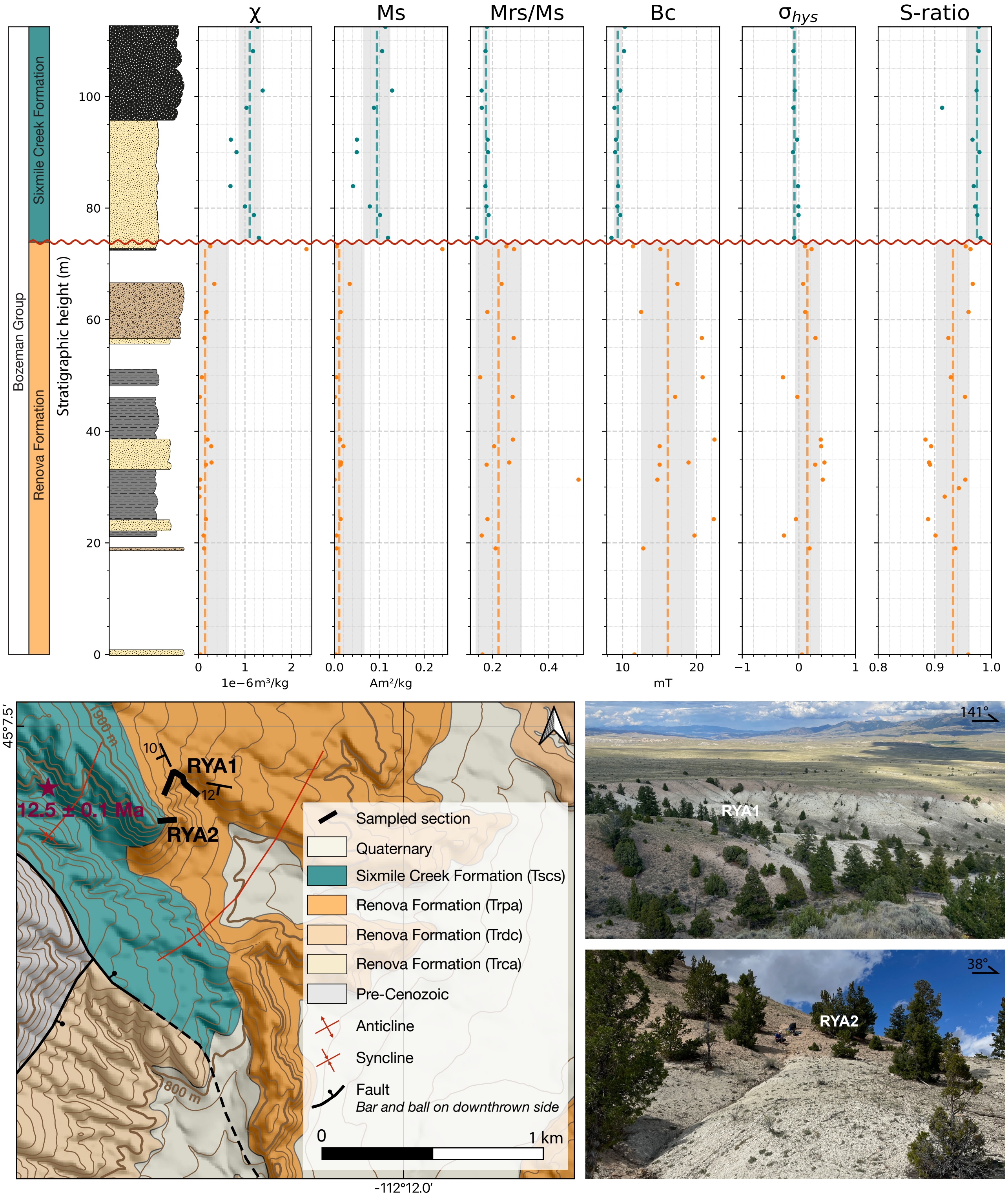

Fig. S4. Overview of the RYA section. Top: Simplified sedimentary log (legend in fig. S1) and rock magnetic properties. From left to right: magnetic susceptibility (χ), saturation magnetization (Ms), saturation remanent magnetization (Mrs) divided by Ms, coercive force (Bc), hysteresis shape parameter (σ_{hys}), and hematite to magnetite concentration (S-ratio). Orange and teal points represent samples taken from the Renova Formation (N = 18) and Sixmile Creek Formation (N = 10), respectively. Dashed orange and teal lines indicate their median values (gray shaded regions 1σ uncertainty). Curvy red line represents the early Miocene unconformity (EMU). Bottom left: Geologic map, with red stars indicating locations of U-Pb ages with 2σ uncertainties (*90*). Bottom right: Field photographs; notice the well-defined difference in lithologic color.

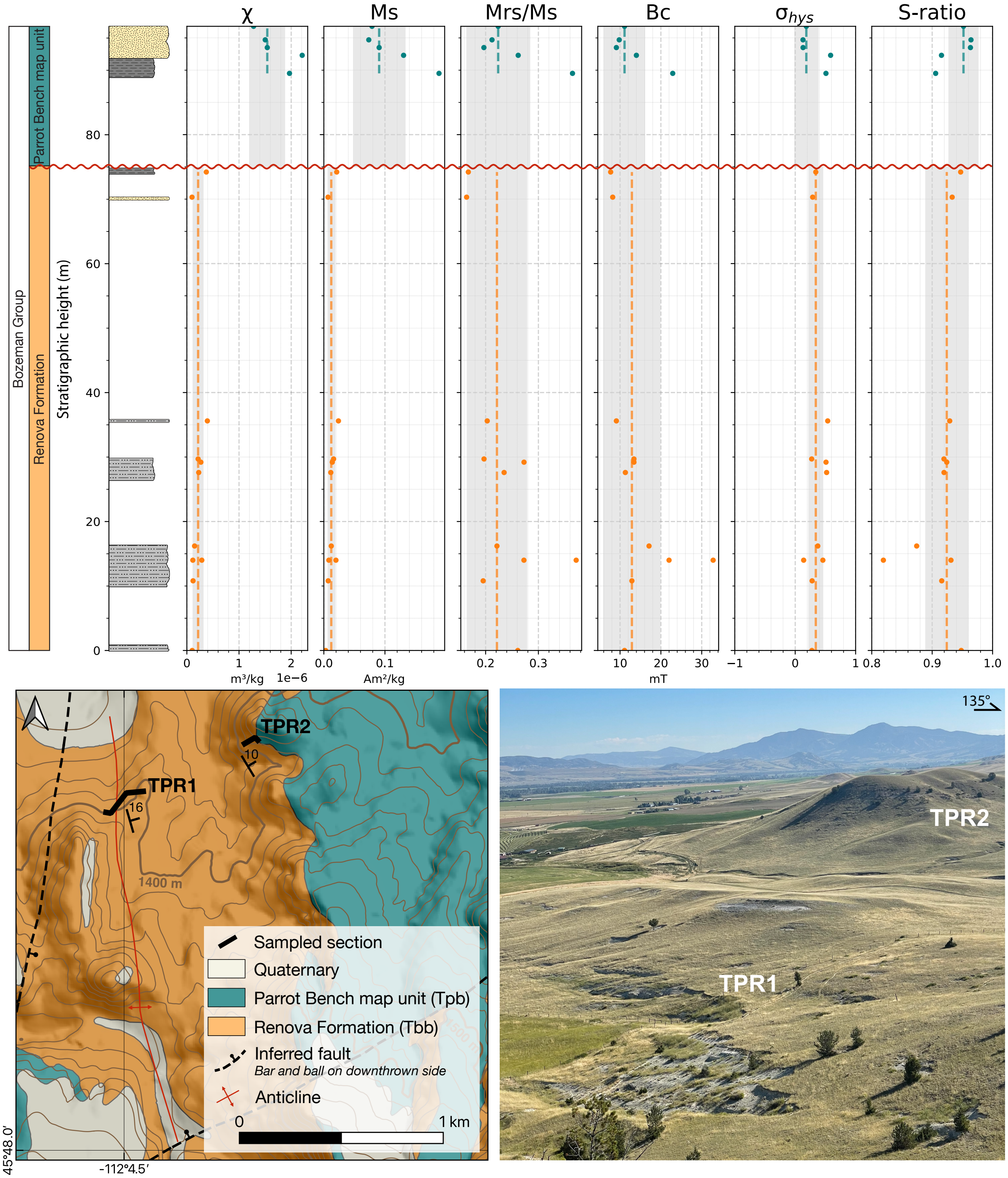

Fig. S5. Overview of the TPR section. Top: Simplified sedimentary log (legend in fig. S1) and rock magnetic properties. From left to right: magnetic susceptibility (χ), saturation magnetization (Ms), saturation remanent magnetization (Mrs) divided by Ms, coercive force (Bc), hysteresis shape parameter (σ_{hys}), and hematite to magnetite concentration (S-ratio). Dashed orange and teal points represent samples taken from the Renova Formation (N = 11) and Sixmile Creek Formation (N = 5), respectively. Orange and teal lines indicate their median values (gray shaded regions 1σ uncertainty). Curvy red line represents the early Miocene unconformity (EMU). Bottom left: Geologic map (*91*). Bottom right: Field photograph.

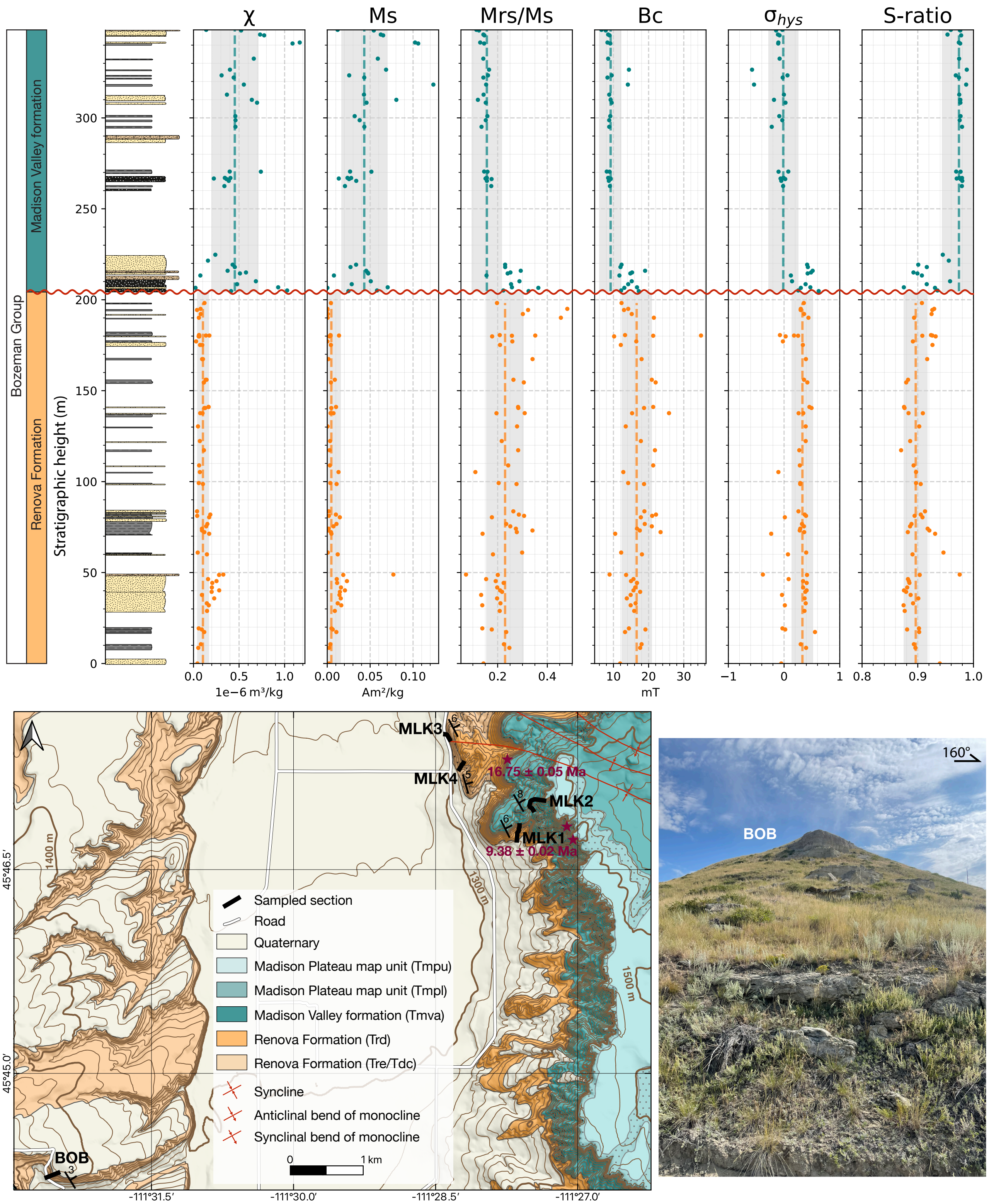

Fig. S6. Overview of the MLK and BOB sections. Top: Simplified sedimentary log (legend in fig. S1) and rock magnetic properties. From left to right: magnetic susceptibility (χ), saturation magnetization (Ms), saturation remanent magnetization (Mrs) divided by Ms, coercive force (Bc), hysteresis shape parameter (σ_{hys}), and hematite to magnetite concentration (S-ratio). Orange and teal points represent samples taken from the Renova Formation (N = 65) and Sixmile Creek Formation (N = 39), respectively. Dashed orange and teal lines indicate their median values (gray shaded regions 1σ uncertainty). Curvy red line represents the early Miocene unconformity (EMU). Bottom left: Geologic map of the MLK (*75*) and BOB (*76*) sections. Red stars indicate locations of U-Pb ages with 2σ uncertainties (*95*). Bottom right: Field photograph.

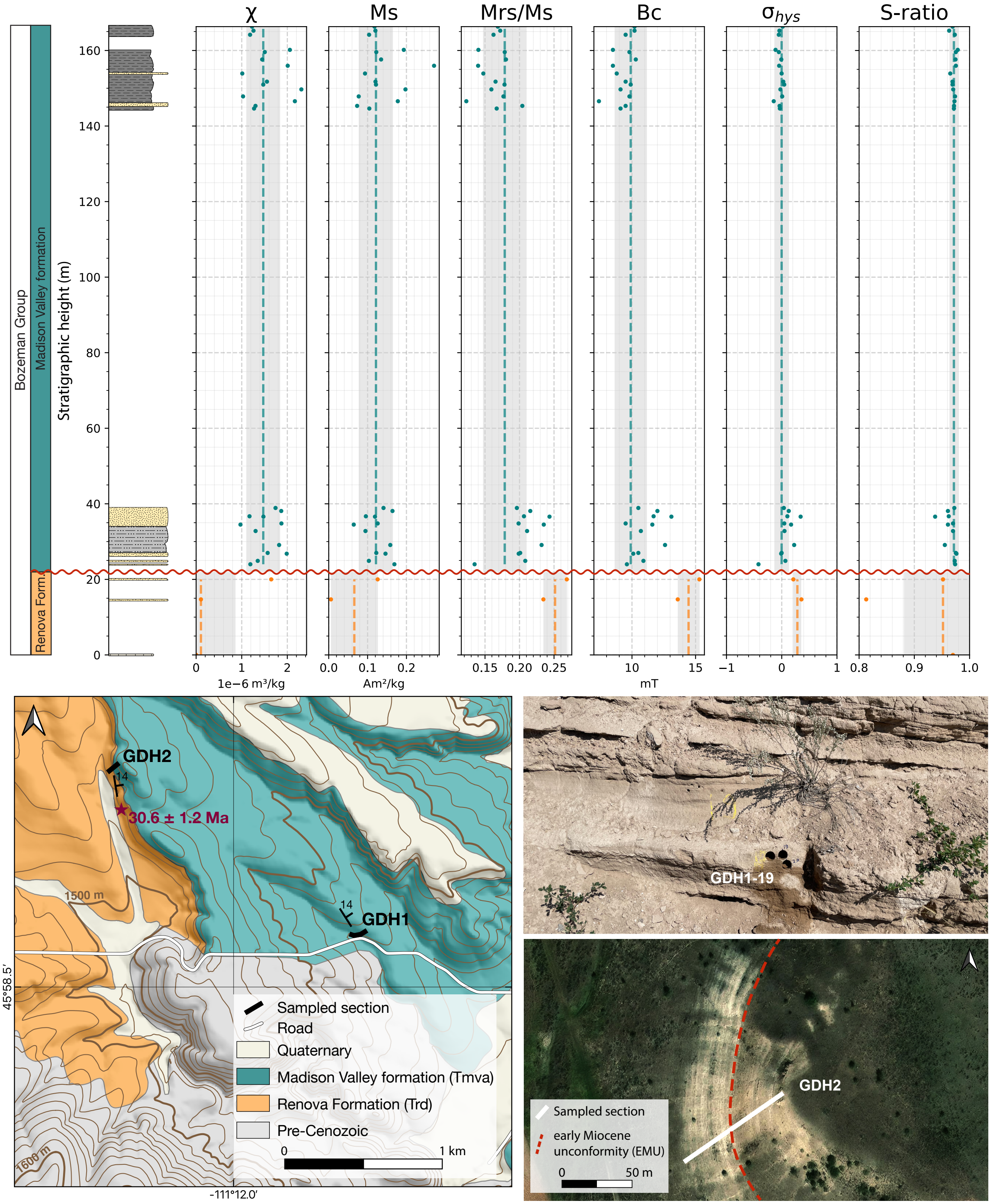

Fig. S7. Overview of the GDH section. Top: Simplified sedimentary log (legend in fig. S1) and rock magnetic properties. From left to right: magnetic susceptibility (χ), saturation magnetization (Ms), saturation remanent magnetization (Mrs) divided by Ms, coercive force (Bc), hysteresis shape parameter (σ_{hys}), and hematite to magnetite concentration (S-ratio). Orange and teal points represent samples taken from the Renova Formation (N = 3) and Sixmile Creek Formation (N = 27), respectively. Dashed orange and teal lines indicate their median values (gray shaded regions 1σ uncertainty). Curvy red line represents the early Miocene unconformity (EMU). Bottom left: Geologic map; adjusted from ref. (*75*) by projecting the observed position of the EMU on the map, assuming a constant bedding attitude of dip direction = 62°N and dip = 14°. Approximate location of K–Ar age indicated by the red star (*77*). Right: Field photograph. Bottom right: Satellite image (*99*).

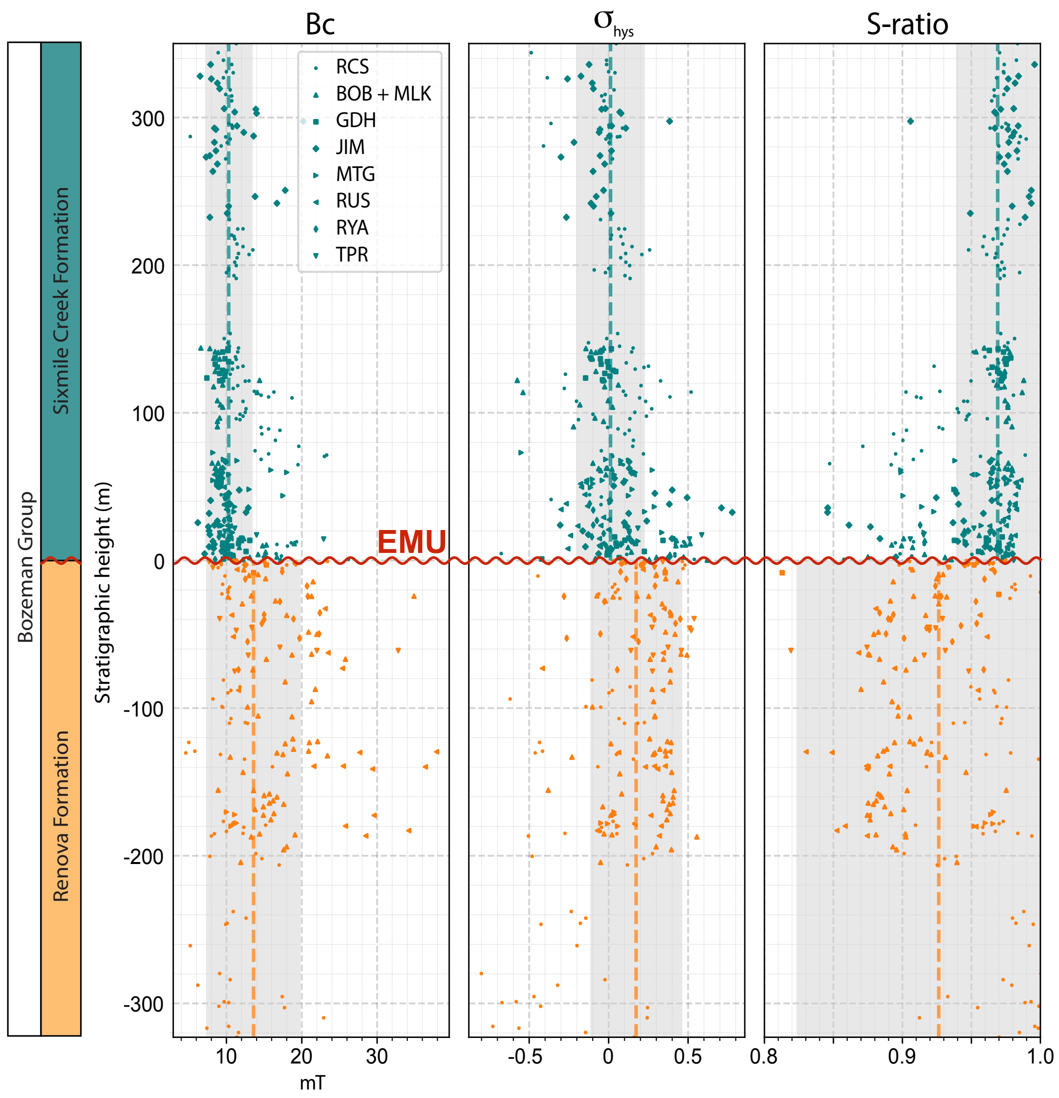

Fig. S8. Additional rock magnetic properties of the eight sections. Sections aligned at the early Miocene unconformity (EMU; curvy red line) positioned at 0 m. From left to right: coercive force (Bc), hysteresis shape parameter (σ_{hys}), and S-ratio. Orange and teal points represent samples taken from the Renova Formation (N = 123) and Sixmile Creek Formation (N = 175), respectively. Dashed orange and teal lines indicate median values; shaded regions, 1σ uncertainty.

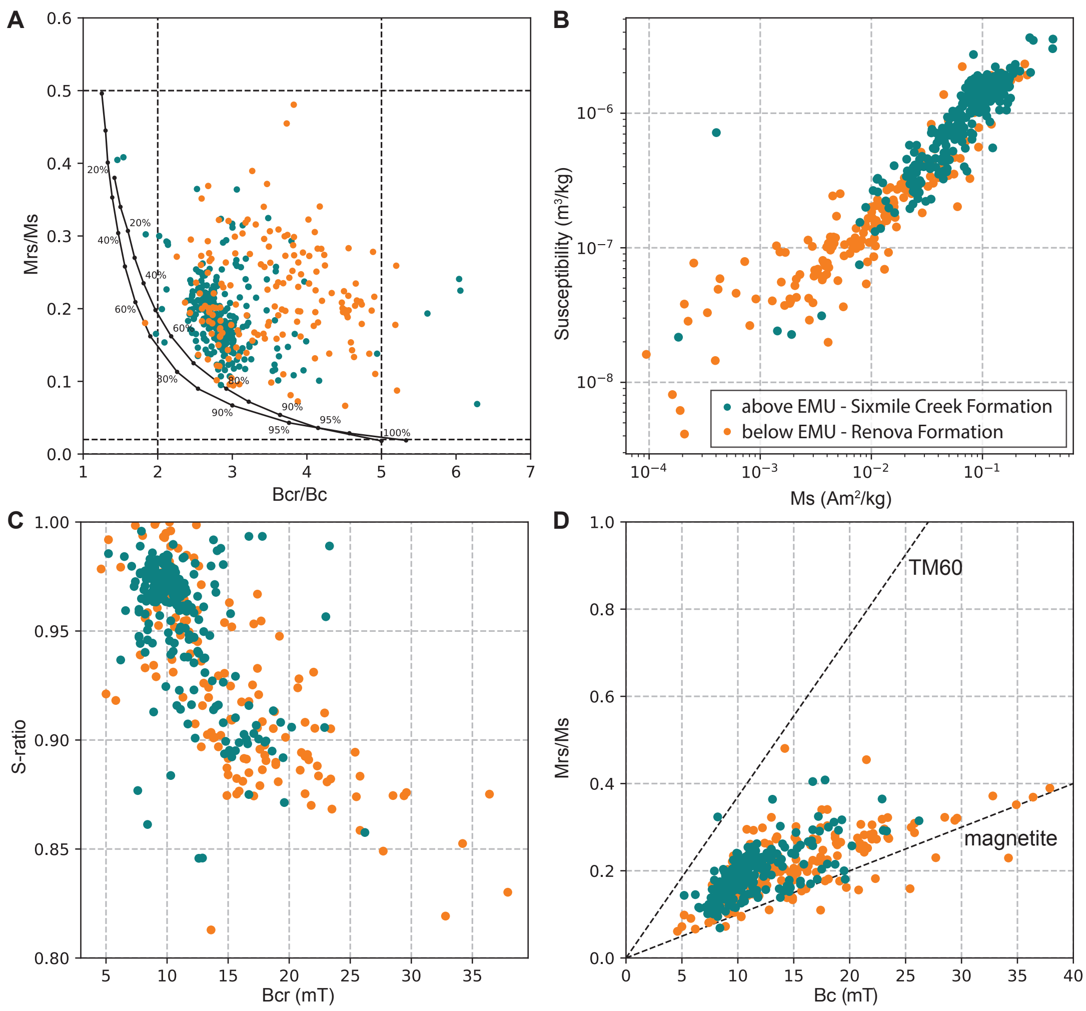

Fig. S9. Rock magnetic diagrams. (**A**) Day diagram (*100*) with the SD + MD mixing curves of Dunlop (*101*). (**B**) Susceptibility versus the saturation magnetization (Ms). (**C**) Coercivity of remanence (Bcr) versus the S-ratio. (**D**) Néel diagram (*102*) with the trend lines for magnetite and titanomagnetite with x = 0.6 (TM60) of Wang and Van der Voo (*103*). Orange and teal points represent samples taken from the Renova Formation (N = 123) and Sixmile Creek Formation (N = 175), respectively.

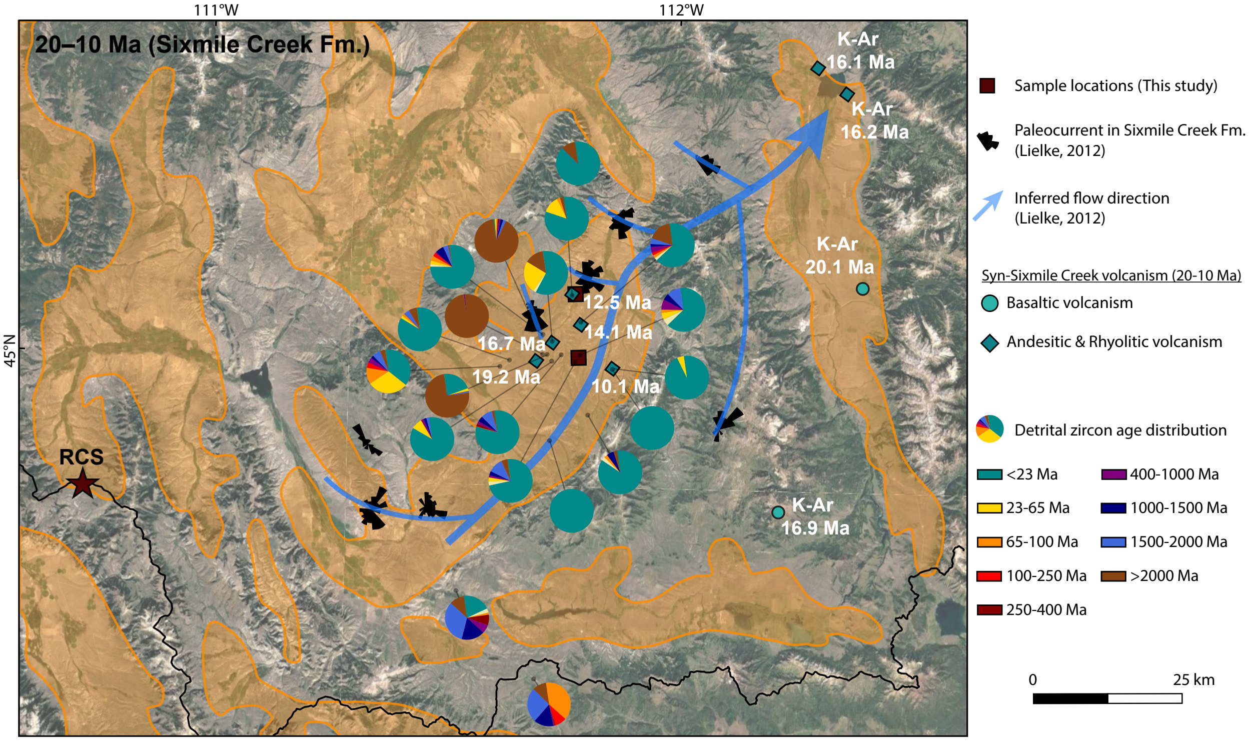

Fig. S10. Zoom-in of Ruby Basin data. Detrital zircon pie charts (*37, 53, 90*) and paleocurrent rose diagrams (*42*) from the Sixmile Creek Formation. Miocene volcanic K–Ar ages (*21*) and U-Pb ages (*22, 23, 90*). Orange basin outlines from Vuke (*12*). Satellite image from Environmental Systems Research Institute (*99*).

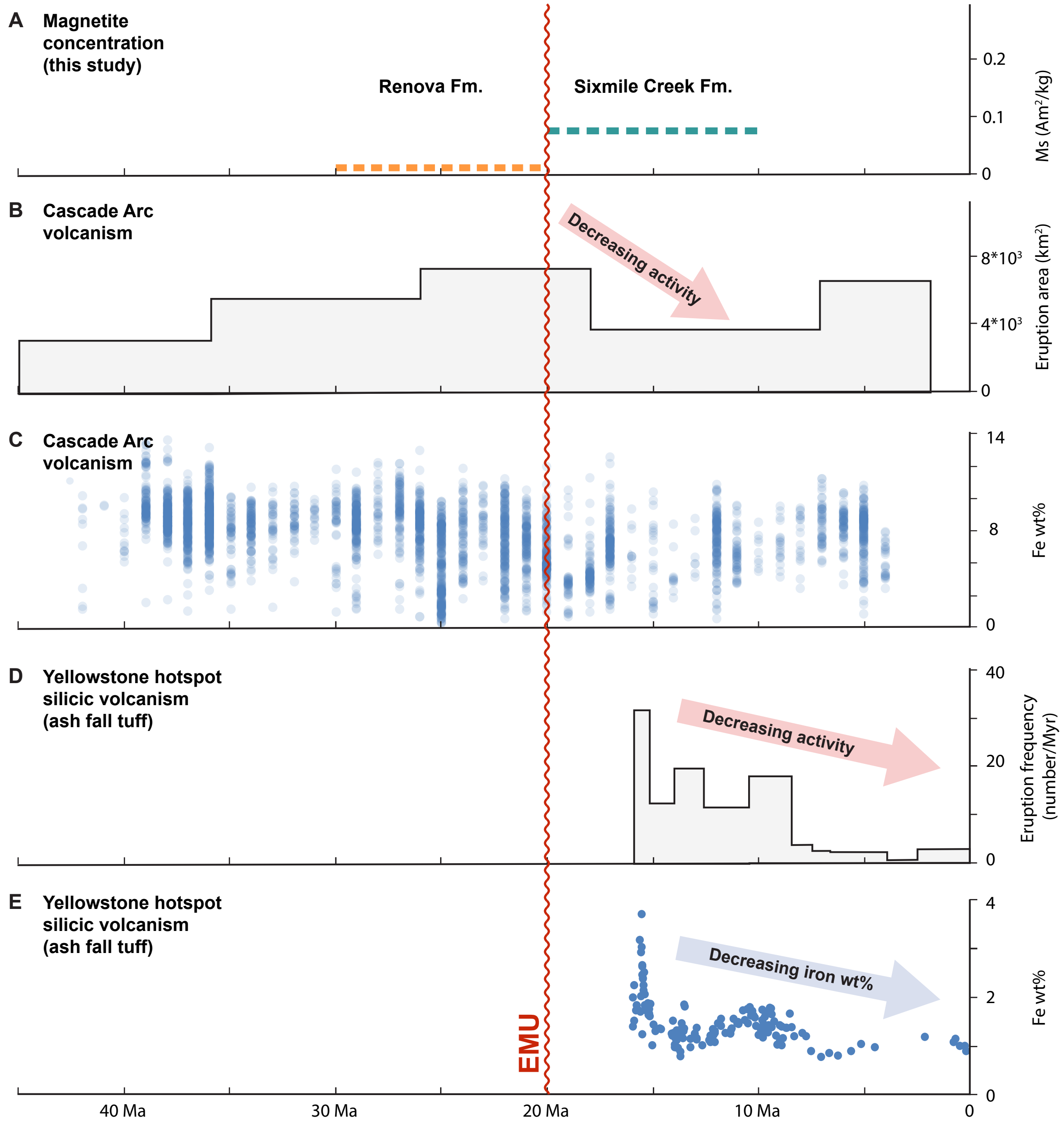

Fig. S11. Overview of volcanic activity 45–0 Ma. (**A**) Median magnetite concentration (Ms = saturation magnetization) from southwestern Montana. (**B**) Eruption activity and (**C**) iron concentration related to Cascade Arc volcanism (*47*). (**D**) Activity and (**E**) iron concentration of Yellowstone hotspot silicic volcanism (*51*). EMU = early Miocene unconformity.

Data S1. (separate file) GPS coordinates. Date, latitude, longitude, and elevation from handheld GPS measurements, visually estimated stratigraphy within each subsection, and projected stratigraphic position of each sample. A comment was added in case we diverged from the standard method of projecting the GPS latitude, longitude, and elevation of each sample with the average bedding.

Data S2. (separate file) Bedding measurements and rock magnetic parameters.

Data S3. (separate file) Detrital zircon samples and their references.

REFERENCES AND NOTES

1. K. N. Constenius, R. P. Esser, P. W. Layer, in *Cenozoic Systems of the Rocky Mountain Region*, R. G. Raynolds, R. M. Flores, Eds. (Rocky Mountain SEPM, 2003), pp. 303–353.

2. P. G. DeCelles, Late Jurassic to Eocene evolution of the Cordilleran thrust belt and foreland basin system, western U.S.A. *Am. J. Sci.* **304**, 105–168 (2004).

3. K. N. Constenius, Late Paleogene extensional collapse of the Cordilleran foreland fold and thrust belt. *GSA Bull.* **108**, 20–39 (1996).

4. W. R. Dickinson, The Basin and Range province as a composite extensional domain. *Int. Geol. Rev.* **44**, 1–38 (2002).

5. S. U. Janecke, Cenozoic extensional processes and tectonics in the northern Rocky Mountains: Southwest Montana and eastern Idaho. *NW Geology* **36**, 111–132 (2007).

6. J. W. Sears, M. S. Hendrix, R. C. Thomas, W. J. Fritz, Stratigraphic record of the Yellowstone hotspot track, Neogene Sixmile Creek Formation grabens, southwest Montana. *J. Volcanol. Geotherm. Res.* **188**, 250–259 (2009).

7. D. L. Rasmussen, *Cenozoic Systems of the Rocky Mountain Region*, R. G. Raynolds, R. M. Flores, Eds. (Rocky Mountain Section SEPM, 2003), pp. 459–477.

8. J. A. Finarelli, C. Badgley, Diversity dynamics of Miocene mammals in relation to the history of tectonism and climate. *Proc. R. Soc. B. Biol. Sci.* **277**, 2721–2726 (2010).

9. M. J. Kohn, T. J. Fremd, Miocene tectonics and climate forcing of biodiversity, western United States. *Geology* **36**, 783–786 (2008).

10. A. D. Barnosky, Distinguishing the effects of the Red Queen and Court Jester on Miocene mammal evolution in the northern Rocky Mountains. *J. Vertebr. Paleontol.* **21**, 172–185 (2001).

11. A. D. Barnosky, M. A. Carrasco, Effects of Oligo-Miocene global climate changes on mammalian species richness in the northwestern quarter of the USA. *Evol. Ecol. Res.* **4**, 811–841 (2002).

12. S. M. Vuke, *Geology of Montana Volume I—Geologic History* (Montana Bureau of Mines and Geology Special Publication, 2020), vol. 122.

13. T. M. Schwartz, R. K. Schwartz, Paleogene postcompressional intermontane basin evolution along the frontal Cordilleran fold-and-thrust belt of southwestern Montana. *GSA Bull.* **125**, 961–984 (2013).

14. D. Gerritsen, S. A. Gilder, A. L. Ludat, M. R. Wack, Magnetostratigraphy and source characterization across the early Miocene unconformity, northern Rocky Mountains, USA. *Terra Nova* **0**, 1–8 (2025).

15. C. P. Chamberlain, H. T. Mix, A. Mulch, M. T. Hren, M. L. Kent-Corson, S. J. Davis, T. W. Horton, S. A. Graham, The Cenozoic climatic and topographic evolution of the western North American Cordillera. *Am. J. Sci.* **312**, 213–262 (2012).

16. J. D. Lonn, R. F. Burmester, R. S. Lewis, M. D. McFaddan, *Geology of Montana Volume I—Geologic History* (Montana Bureau of Mines and Geology Special Publication, 2020), vol. 122.

17. K. E. Barton, D. G. Howell, J. F. Vigil, The North America tapestry of time and terrain (No. 2781) U.S. Geological Survey, scale 1:8,000,000 (2003); https://pubs.usgs.gov/imap/i2781/.

18. T. A. Harms, J. A. Baldwin, *Geology of Montana Volume I—Geologic History* (Montana Bureau of Mines and Geology Special Publication, 2020), vol. 122.

19. K. C. Scarberry, P. V. Yakovlev, T. M. Schwartz, *Geology of Montana Volume I—Geologic History* (Montana Bureau of Mines and Geology Special Publication, 2020), vol. 122.

20. S. S. Harlan, 40Ar/39Ar dates from alkaline intrusions in the northern Crazy Mountains, Montana: Implications for the timing and duration of alkaline magmatism in the central Montana alkalic province. *Rocky Mt. Geol.* **41**, 45–55 (2006).

21. W. J. Fritz, J. W. Sears, J. M. Wampler, Cenozoic volcanic rocks of southwestern Montana. *NW Geol.* **36**, 91–110 (2007).

22. J. G. Mosolf, Geologic field guide to the tertiary volcanic rocks in the Elliston 30′ × 60′ quadrangle, west-central Montana. *NW Geol.* **44**, 213–232 (2015).

23. J. G. Mosolf, Geologic map of the Virginia City 7.5′ quadrangle, Madison County, Montana: Montana bureau of mines and geology geologic map 80, 1 sheet, scale 1:24,000 (2021); https://mbmg.mtech.edu/mbmgcat/public/ListCitation.asp?pub_id=32390&.

24. S. U. Janecke, Sedimentation and paleogeography of an Eocene to Oligocene rift zone, Idaho and Montana. *GSA Bulletin* **106**, 1083–1095 (1994).

25. E. T. Ruppel, Cenozoic block uplifts in east-central Idaho and southwest Montana: Geological Survey Professional Paper 1224 (1982), 24 p.

26. M. W. Reynolds, *Basin and Range Symposium and Field Conference Proceedings*, G. W. Newman, H. D. Goodge, Eds. (Rocky Mountain Association of Geologists and Utah Geological Association, 1979), pp. 185–193.

27. W. D. Kuenzi, R. W. Fields, Tertiary stratigraphy, structure, and geologic history, Jefferson Basin, Montana. *GSA Bull.* **82**, 3373–3394 (1971).

28. R. W. Fields, A. R. Tabrum, R. Nichols, in *Cenozoic Paleogeography of West-Central United States*, R. M. Flores, S. S. Kaplan, Eds. (Rocky Mountain Section, Society of Economic and Petroleum Geologists, 1985), pp. 9–36.

29. D. L. Hanneman, C. J. Wideman, Sequence stratigraphy of Cenozoic continental rocks, southwestern Montana. *GSA Bulletin* **103**, 1335–1345 (1991).

30. D. L. Hanneman, C. J. Wideman, *Paleoenvironmental Record and Applications of Calcretes and Palustrine Carbonates*, A. M. Alonzo-Zarza, L. H. Tanner, Eds. (Geological Society of America Special Paper 416, 2006), pp. 1–15.

31. D. L. Hanneman, C. J. Wideman, *Carbonates in Continental Settings: Geochemistry, Diagenesis, and Applications*, A. M. Alonzo-Zarza, L. H. Tanner, Eds. (Elsevier, 2010), vol. 62, chap. 5, pp. 215–273.

32. R. C. Thomas, J. W. Sears, *Geology of Montana Volume I—Geologic History* (Montana Bureau of Mines and Geology Special Publication, 2020), vol. 122.

33. S. U. Janecke, C. J. VanDenburg, J. J. Blankenau, J. W. M'Gonigle, Long-distance longitudinal transport of gravel across the Cordilleran thrust belt of Montana and Idaho. *Geology* **28**, 439–442 (2000).

34. R. A. Portner, M. S. Hendrix, J. C. Stalker, D. P. Miggins, S. D. Sheriff, Sedimentary response to orogenic exhumation in the northern rocky mountain basin and range province, flint creek basin, west-central Montana. *Can. J. Earth Sci.* **48**, 1131–1154 (2011).

35. C. N. Stroup, P. K. Link, C. M. Fanning, Provenance of late Miocene fluvial strata of the Sixmile Creek Formation, southwest Montana: Evidence from detrital zircon. *NW Geol.* **37**, 69–84 (2008).

36. S. U. Janecke, R. J. Dorsey, J. Kickham, J. P. Matoush, W. McIntosh, Geologic Map of the Bachelor Mountain 7.5′ Quadrangle Beaverhead County, Montana: Montana Bureau of Mines Open File Report 525, 27 p., 1 sheet, scale 1:24,000 (2005); https://mbmg.mtech.edu/pdf-open-files/mbmg525-BachelorMtn.pdf.

37. L. M. Staisch, J. E. O'Connor, C. M. Cannon, C. Holm-Denoma, P. K. Link, J. Lasher, J. A. Alexander, Major reorganization of the Snake River modulated by passage of the Yellowstone Hotspot. *GSA Bulletin* **134**, 1834–1844 (2022).

38. C. G. Elliott, C. McDonald, Geologic map and geohazard assessment of Silver Bow County, Montana, Montana Bureau of Mines and Geology Open-File Report 585, 88 p., 3 sheets, scale 1:50,000 (2009); https://mbmg.mtech.edu/pdf-open-files/mbmg585_silverbowcounty.pdf.

39. G. J. Retallack, Refining a pedogenic-carbonate CO_2 paleobarometer to quantify a middle Miocene greenhouse spike. *Palaeogeogr. Palaeoclimatol. Palaeoecol.* **281**, 57–65 (2009).

40. K. Methner, A. Mulch, J. Fiebig, E. Krsnik, N. Löffler, D. Bajnai, C. P. Chamberlain, Warm high-elevation mid-latitudes during the Miocene climatic optimum: Paleosol clumped isotope temperatures from the northern Rocky Mountains, USA. *Paleoceanogr. Paleoclimatol.* **36**, e2020PA003991 (2021).

41. E. B. Harris, M. J. Kohn, C. A. E. Strömberg, Stable isotope compositions of herbivore teeth indicate climatic stability leading into the mid-Miocene Climatic Optimum, in Idaho, U.S.A. *Palaeogeogr. Palaeoclimatol. Palaeoecol.* **546**, 109610 (2020).

42. K. J. Lielke, "The climatic, biotic and tectonic evolution of the Paleogene Renova formation of southwestern Montana," thesis, University of Montana (2012).

43. T. M. Schwartz, R. K. Schwartz, A. L. Weislogel, Orogenic recycling of detrital zircons characterizes age distributions of North American Cordilleran strata. *Tectonics* **38**, 4320–4334 (2019).

44. J. L. Rothfuss, K. Lielke, A. L. Weislogel, *Mineralogical and Geochemical Approaches to Provenance: Geological Society of America Special Paper 487*, E. T. Rasbury, S. R. Hemming, N. R. Riggs, Eds. (2012), pp. 63–95.

45. P. K. Link, C. M. Fanning, L. P. Beranek, Reliability and longitudinal change of detrital-zircon age spectra in the Snake River system, Idaho and Wyoming: An example of reproducing the bumpy barcode. *Sediment. Geol.* **182**, 101–142 (2005).

46. G. R. Priest, Volcanic and tectonic evolution of the Cascade Volcanic Arc, central Oregon. *J. Geophys. Res.* **95**, 19583–19599 (1990).

47. E. A. du Bray, D. A. John, Petrologic, tectonic, and metallogenic evolution of the Ancestral Cascades magmatic arc, Washington, Oregon, and northern California. *Geosphere* **7**, 1102–1133 (2011).

48. J. G. Smith, Geologic map of upper Eocene to Holocene volcanic and related rocks in the Cascade Range, Washington: U.S. Geological Survey Miscellaneous Investigations Map I-2005, 20 p., 2 sheets, scale 1:500,000 (1993); https://pubs.usgs.gov/imap/2005/.

49. D. R. Sherrod, J. G. Smith, Geologic map of upper Eocene to Holocene volcanic and related rocks of the Cascade Range, Oregon: U.S. Geological Survey Miscellaneous Investigations Map I-2569, 17 p., 2 sheets, scale 1:500,000 (2000); https://pubs.usgs.gov/imap/i-2569/.

50. B. P. Nash, M. E. Perkins, J. N. Christensen, D.-C. Lee, A. N. Halliday, The Yellowstone hotspot in space and time: Nd and Hf isotopes in silicic magmas. *Earth Planet. Sci. Lett.* **247**, 143–156 (2006).

51. M. E. Perkins, B. P. Nash, Explosive silicic volcanism of the Yellowstone hotspot: The ash fall tuff record. *GSA Bulletin* **114**, 367–381 (2002).

52. K. Nielsen, R. C. Thomas, Paleoenvironmental reconstruction of the mid-Miocene Beaverhead Graben in southwest Montana. *Geological Society of America Abstracts with Programs* **36**, 546 (2004).

53. J. G. Mosolf, D. T. Brennan, A. Kylander-Clark, U-Pb geochronology data from rock samples collected in the Dillon, Ennis, Gardiner, Hamilton, Hebgen Lake, Lima, and Wisdom 30′ x 60′ quadrangles, western Montana, 2022–2023: Montana Bureau of Mines and Geology Analytical Dataset 5 (2023); https://mbmg.mtech.edu/mbmgcat/public/ListCitation.asp?pub_id=32596&.

54. W. J. Fritz, J. W. Sears, Tectonics of the Yellowstone hotspot wake in southwestern Montana. *Geology* **21**, 427–430 (1993).

55. A. Byerly, B. Tikoff, M. Kahn, B. Jicha, R. Gaschnig, A. K. Fayon, Internal fabrics of the Idaho batholith, USA. *Lithosphere* **9**, 283–298 (2016).

56. D. Foster, P. A. Mueller, A. Heatherington, J. N. Gifford, T. J. Kalakay, Lu–Hf systematics of magmatic zircons reveal a Proterozoic crustal boundary under the Cretaceous Pioneer batholith, Montana. *Lithos* **142–143**, 216–225 (2012).

57. S. M. Vuke, K. W. Porter, J. D. Lonn, D. A. Lopez, Geologic map of Montana: Montana Bureau of Mines and Geology Geologic Map 62, 73 p., 2 sheets, scale 1:500,000 (2007); https://ngmdb.usgs.gov/Prodesc/proddesc_81651.htm.

58. V. E. Camp, J. A. Wolff, Spatio-temporal evolution of the main-phase Columbia River Basalt Group and its genetic link to the Yellowstone hotspot. *GSA Bulletin* **137**, 3317–3339 (2025).

59. E. A. Mankinen, M. Prévot, C. S. Grommé, R. S. Coe, The Steens Mountain (Oregon) geomagnetic polarity transition: 1. Directional history, duration of episodes, and rock magnetism. *J. Geophys. Res. Solid Earth* **90**, 10393–10416 (1985).

60. M. A. Coble, G. A. Mahood, Initial impingement of the Yellowstone plume located by widespread silicic volcanism contemporaneous with Columbia River flood basalts. *Geology* **40**, 655–658 (2012).

61. E. B. Cahoon, M. J. Streck, A. A. Koppers, D. P. Miggins, Reshuffling the Columbia River Basalt chronology—Picture Gorge Basalt, the earliest- and longest-erupting formation. *Geology* **48**, 348–352 (2020).

62. L. M. Chetel, S. U. Janecke, A. R. Carroll, B. L. Beard, C. M. Johnson, B. S. Singer, Paleogeographic reconstruction of the Eocene Idaho River, North American Cordillera. *GSA Bulletin* **123**, 71–88 (2011).

63. J. W. Sears, P. C. Ryan, *Cenozoic Systems of the Rocky Mountain Region*, R. G. Raynolds, R. M. Flores, Eds. [Rocky Mountain Section (SEPM), 2003].

64. A. L. Anderson, Drainage diversion in the northern Rocky Mountains of east-central Idaho. *J. Geol.* **55**, 61–75 (1947).

65. R. Portner, M. Hendrix, Preliminary geologic map of the eastern Flint Creek Basin west-central Montana: Montana Bureau of Mines and Geology Open-File Report 521, 17 p., 1 sheet, scale 1:24,000 (2005); https://mbmg.mtech.edu/pdf-open-files/mbmg521-FlintCrkBasin.pdf.

66. T. M. Schwartz, K. Methner, A. Mulch, S. A. Graham, C. P. Chamberlain, Paleogene topographic and climatic evolution of the Northern Rocky Mountains from integrated sedimentary and isotopic data. *GSA Bulletin* **131**, 1203–1223 (2019).

67. M. C. Stickney, M. J. Bartholomew, Seismicity and late Quarternary faulting of the northern Basin and Range Province, Montana and Idaho. *SSA Bulletin* **77**, 1602–1625 (1987).

68. D. Schmeelk, R. Bendick, M. Stickney, C. Bomberger, Kinematic evidence for the effect of changing plate boundary conditions on the tectonics of the northern U.S. Rockies. *Tectonics* **36**, 1090–1102 (2017).

69. C. J. Vandenburg, S. U. Janecke, W. C. McIntosh, Three-dimensional strain produced by >50 My of episodic extension, Horse Prairie basin area, SW Montana, U.S.A. *J. Struct. Geol.* **20**, 1747–1767 (1998).

70. A. M. Friedrich, H. P. Bunge, S. M. Rieger, L. Colli, S. Ghelichkhan, R. Nerlich, Stratigraphic framework for the plume mode of mantle convection and the analysis of interregional unconformities on geological maps. *Gondwana Res.* **53**, 159–188 (2018).

71. M. Hoggard, J. Austermann, C. Randel, S. Stephenson, *Mantle Convection and Surface Expressions*, H. Marquardt, J. Konter, M. Ballmer, S. Cottaar, Eds. [American Geophysical Union (AGU), 2021], pp. 371–411.

72. R. W. Griffiths, I. H. Campbell, Interaction of mantle plume heads with the Earth's surface and onset of small-scale convection. *J. Geophys. Res.* **96**, 18295 (1991)

73. B. Steinberger, P. L. Nelson, S. P. Grand, W. Wang, Yellowstone plume conduit tilt caused by large-scale mantle flow. *Geochem. Geophys. Geosyst.* **20**, 5896–5912 (2019).

74. L. Liu, D. R. Stegman, Origin of Columbia River flood basalt controlled by propagating rupture of the Farallon slab. *Nature* **482**, 386–389 (2012).

75. S. M. Vuke, Geology of western and northern Gallatin Valley, southwestern Montana Montana Bureau of Mines and Geology Open-File Report 481, 40 p., 1 sheet, scale 1:50,000 (2003); https://mbmg.mtech.edu/mbmgcat/public/ListCitation.asp?pub_id=11352&.

76. S. M. Vuke, Geologic map of the Cenozoic deposits of the lower Jefferson Valley, southwestern Montana: Montana Bureau of Mines and Geology Open-File Report 537, 41 p., 1 sheet, scale 1:50,000 (2006); https://mbmg.mtech.edu/pdf-open-files/mbmg537_lowerjefferson.pdf.

77. G. C. Hughes, "Cenozoic geology and geomorphology of the Dry Creek Valley, Gallatin County, Montana," thesis, Montana State University 1980).

78. G. A. Paterson, X. Zhao, M. Jackson, D. Heslop, Measuring, processing, and analyzing hysteresis data. *Geochem. Geophys. Geosyst.* **19**, 1925–1945 (2018).

79. K. Fabian, Some additional parameters to estimate domain state from isothermal magnetization measurements. *Earth Planet. Sci. Lett.* **213**, 337–345 (2003).

80. J. Bloemendal, J. W. King, F. R. Hall, S.-J. Doh, Rock magnetism of late Neogene and Pleistocene deep-sea sediments: Relationship to sediment source, diagenetic processes, and sediment lithology. *J. Geophys. Res.* **97**, 4361–4375 (1992).

81. NOAA, ETOPO 2022 15 Arc-Second Global Relief Model: NOAA National Centers for Environmental Information (2022); https://ncei.noaa.gov/products/etopo-global-relief-model.

82. A. D. Barnosky, M. Holmes, R. Kirchholtes, E. Lindsey, K. C. Maguire, A. W. Poust, M. A. Stegner, J. Sunseri, B. Swartz, J. Swift, N. A. Villavicencio, G. O. U. Wogan, Prelude to the Anthropocene: Two new North American land mammal ages (NALMAs). *Anthr. Rev.* **1**, 225–242 (2014).

83. J. W. M'Gonigle, Geologic Map of the Deadman Pass Quadrangle, Beaverhead County Montana, and Lemhi County, Idaho: U.S. Geological Survey, scale 1:24,000 (1994); https://pubs.usgs.gov/publication/gq1753.

84. E. B. Harris, C. A. E. Strömberg, N. D. Sheldon, S. Y. Smith, M. Ibañez-Mejia, Revised chronostratigraphy and biostratigraphy of the early-middle Miocene Railroad Canyon section of central-eastern Idaho, USA. *GSA Bulletin* **129**, 1241–1251 (2017).

85. A. D. Barnosky, F. Bibi, S. S. B. Hopkins, R. Nichols, Biostratigraphy and magnetostratigraphy of the mid-Miocene Railroad Canyon Sequence, Montana and Idaho, and age of the mid-Tertiary unconformity west of the continental divide. *J. Vertebr. Paleontol.* **27**, 204–224 (2007).

86. J. J. M. Calede, Pattern and processes of the mammalian turnover of the Arikareean in the northern Rocky Mountains. *J. Vertebr. Paleontol.* **40**, e1767117 (2020).

87. J. G. Mosolf, S. M. Vuke, Geologic map of the Gravely Mountain 7.5′ quadrangle, Powell County, Montana: Montana Bureau of Mines and Geology Open-File Report 693, 1 sheet, scale 1:24,000 (2017).

88. J. J. Calede, D. L. Rasmussen, Field guide to the geology and paleontology of the Cabbage Patch beds in the Flint Creek Basin (Renova Formation, Arikareean). *NW Geol.* **44**, 157–188 (2015).

89. S. M. Vuke, Geologic map of the Divide area, southwest Montana: Montana Bureau of Mines and Geology Open-File Report 502, 36 p., 1 sheet, scale 1:50,000 (2004); https://mbmg.mtech.edu/mbmgcat/public/ListCitation.asp?pub_id=11373&.

90. D. T. Brennan, J. W. Sears, J. G. Mosolf, Geologic map of the Belmont Park Ranch 7.5′ quadrangle, Madison County, Montana: Montana Bureau of Mines and Geology Geologic Map 99, 1 sheet, scale 1:24,000 (2024); https://mbmg.mtech.edu/mbmgcat/public/ListCitation.asp?pub_id=32719&.

91. S. M. Vuke, W. W. Coppinger, B. E. Cox, Geologic map of Cenozoic deposits in the Upper Jefferson Valley, southwestern Montana: Montana Bureau of Mines and Geology Open-File Report 505, 35 p., 1 sheet, scale 1:50,000 (2004); https://mbmg.mtech.edu/mbmgcat/public/ListCitation.asp?pub_id=11376&.

92. P. K. Link, C. M. Fanning, C. N. Stroup, Detrital zircon U-Pb geochronologic data for selected Cretaceous, Paleogene, Neogene, and Holocene sandstones and river sands in southwest Montana and east-central Idaho: Montana Bureau of Mines and Geology Open-File Report 569 (2008); https://mbmg.mtech.edu/pdf-open-files/mbmg569/mbmg569.pdf.

93. R. B. Axelrod, “Tertiary sedimentary facies depositional environments and structure Jefferson Basin southwest Montana,” thesis, University of Montana (1984).

94. X. Wang, B. C. Wideman, R. Nichols, D. L. Hanneman, A new species of Aelurodon (Carnivora, Canidae) from the Barstovian of Montana. *J. Vertbr.* **24**, 445–452 (2004).

95. C. Montejo, J. R. Stanley, D. E. Miller, paper presented at the 73rd Annual Meeting Rocky Mountain Section of The Geological Society of America, Fort Collins, Colorado, USA, 2023.

96. W. P. Roe, “Tertiary sediments of the Big Hole valley and Pioneer Mountains, southwestern Montana: Age, provenance, and tectonic implications,” thesis, The University of Montana, Missoula, MT (2010).

97. A. E. McCafferty, C. A. San, C. Lawley, G. E. Graham, M. G. Gadd, D. L. Huston, K. D. Kelley, S. Paradis, J. M. Peter, K. Czarnota, Magnetic and related derivative GeoTIFF grids and data for the United States and Canada: U.S. Geological Survey data release (2023); https://data.usgs.gov/datacatalog/data/USGS:619a9a3ad34eb622f692f961.

98. J. D. Walker, T. D. Bowers, R. A. Black, A. F. Glazner, G. L. Farmer, R. W. Carlson, *Geoinformatics: Data to Knowledge*, A. K. Sinha, Ed. (Geological Society of America, 2006).

99. Environmental Systems Research Institute, *World Imagery* (2009). https://hub.arcgis.com/maps/esri::world-imagery.

100. R. Day, M. Fuller, V. A. Schmidt, Hysteresis properties of titanomagnetites: Grain-size and compositional dependence. *Phys. Earth Planet. Inter.* **13**, 260–267 (1977).

101. D. J. Dunlop, Theory and application of the Day plot (Mrs/Ms versus Hcr/Hc) 1. Theoretical curves and tests using titanomagnetite data. *J. Geophys. Res. Solid Earth* **107**, EPM-4 (2002).

102. L. Néel, Some theoretical aspects of rock-magnetism. *Adv. Phys.* **4**, 191–243 (1955).

103. D. Wang, R. van der Voo, The hysteresis properties of multidomain magnetite and titanomagnetite/titanomaghemite in mid-ocean ridge basalts. *Earth Planet. Sci. Lett.* **220**, 175–184 (2004).
